# Tailoring Symmetry Breaking in Engineered van der Waals Superlattices

**DOI:** 10.1002/adma.74569

**Published:** 2026-08-11

**Authors:** Keda Jin, Lennart Klebl, Zachary A. H. Goodwin, Junting Zhao, Felix Lüpke, Dante M. Kennes, Jose Martinez‐Castro, Markus Ternes

**Affiliations:** ^1^ Peter Grünberg Institut (PGI‐3) Forschungszentrum Jülich Jülich Germany; ^2^ Jülich Aachen Research Alliance Fundamentals of Future Information Technology Jülich Germany; ^3^ Institut für Experimentalphysik II B RWTH Aachen Universität Aachen Germany; ^4^ Institut für Theoretische Physik und Astrophysik and Würzburg‐Dresden Cluster of Excellence ct.qmat Universität Würzburg Würzburg Germany; ^5^ Department of Materials University of Oxford Oxford United Kingdom; ^6^ John A Paulson School of Engineering and Applied Sciences Harvard University Cambridge USA; ^7^ Institute of Physics II Universität zu Köln Köln Germany; ^8^ Institut für Theorie der statistischen Physik RWTH Aachen Universität Aachen Germany; ^9^ Max Planck Institute for the Structure and Dynamics of Matter Center for Free Electron Laser Science Hamburg Germany

**Keywords:** charge density waves, scanning tunneling microscopy, superlattice engineering, symmetry breaking, van der Waals heterostructures

## Abstract

Superlattice engineering in van der Waals (vdW) heterostructures (e.g., by moiré engineering) provides a powerful platform for designing electronic bands and realizing correlated and topological quantum phenomena.

Here, we pioneer a scheme to tailor superpotentials based on intrinsic substrate electronic orders. We show that this establishes a robust, self‐aligned, and highly versatile route to band‐structure control, as we demonstrate in graphene by engineering two distinct, nearly commensurate superlattices using the charge density waves (CDWs) of 1T‐NbSe_2_. In these superlattices, the graphene's Dirac cones are folded either to the Γ‐point or to the K‐points of the mini‐Brillouin zone (mBZ). Using scanning tunneling microscopy, we observe that the Γ‐folded system preserves $C_{3}$ symmetry, while the K‐folded system exhibits symmetry breaking. Combining density functional theory with an interlayer interaction model, we reveal that this difference is not electronically driven but originates from a structural instability. Our work establishes superlattice engineering for designer quantum states and unveils a structural mechanism for controlled emergent symmetry breaking.

## Graphical abstract

We pioneer a scheme to tailor superpotentials in graphene based on intrinsic substrate electronic orders rather than twist angle. The resulting superpotential folds graphene's Dirac cones to either Γ or K‐points, leading to distinct symmetry behaviors. Experiments and theory reveal that the symmetry breaking arises from structural effects, offering a new route to design electronic states in van der Waals (vdW) heterostructures.

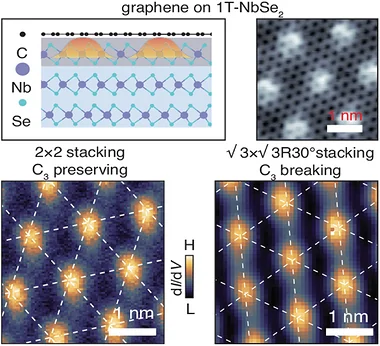

## 1 Introduction

Superlattices in van der Waals (vdW) heterostructures offer a way to reshape the electronic structure of two‐dimensional (2D) materials and stabilize interaction‐driven phases that are not present in the individual constituents [1, 2, 3, 4, 5, 6]. By inducing a long‐wavelength potential on top of the atomic lattice, one can induce band folding, reduce bandwidths, and strengthen electron‐electron interactions [6, 7, 8, 9]. A particularly successful implementation of this concept uses moiré patterns generated by twisting atomically thin layers [3, 10].

This strategy has led to the discovery of many novel phenomena, including correlated insulating states [3, 11], unconventional superconductivity [12, 13, 14], topological states [15, 16], the fractional quantum Hall effect [17, 18], and emergent magnetism [19, 20]. Notably, many of these phenomena involve spontaneous symmetry breaking, highlighting the connection between superlattice geometry and the emergence of new quantum phases.

An alternative approach to engineer superlattices is to use the intrinsic electronic order in one material, such as charge density waves (CDWs), to impose a superlattice potential in an adjacent layer through proximity effects [21, 22, 23]. Unlike moiré engineering via twist angles, where achieving the desired twist angle during fabrication can be challenging, superlattices based on intrinsic order offer an inherent advantage: when the two constituent lattices are nearly commensurate, the system spontaneously relaxes into an energetically favorable registry [24, 25, 26]. This “lock‐in” mechanism, in which the layers self‐align through slight rotations or lateral sliding, yields structures that are both energetically stable and experimentally reproducible [27]. As a result, this type of superlattice engineering provides a robust and deterministic platform for realizing novel quantum phases.

In graphene, the symmetry of the superlattice defines at which points the Dirac cones appear in the mini‐Brillouin zone (mBZ) [28]. As the K and $K^{\prime }$ valleys of graphene are related by time‐reversal symmetry, there are only two distinct folding configurations. In one configuration, both K and $K^{\prime }$ valleys are folded onto the Γ point of the mBZ (Γ‐folding), merging the two Dirac cones in momentum space [29, 30]. In the other configuration, the valley identities are retained, with K and $K^{\prime }$ mapped separately to the $K_{m}$ and $K_{m}^{\prime }$ corners of the mBZ (K‐folding) [28, 31].

While symmetry arguments predict these two folding scenarios, they do not completely determine the resulting electronic structure. Hybridization with substrate bands, interlayer relaxation, and subtle variations in local stacking registry may profoundly reshape the band structure in ways that cannot be anticipated from geometry alone [25, 32]. Moreover, such effects may differ qualitatively between the two configurations: do Γ‐folding and K‐folding produce equivalent electronic reconstructions, or does one geometry harbor instabilities absent in the other? Answering this question requires the combination of experiment and theory within a controlled material system.

Here, we demonstrate deterministic control over the Dirac cone folding by using the CDW in 1T‐NbSe_2_ [33, 34, 35] to engineer two distinct, nearly commensurate superlattices on a graphene layer. We create a 2×2superlattice relative to the CDW lattice that exhibits Γ‐folding, and a $\sqrt{3}\times \;\sqrt{3}\;R30^{\circ }\mathrm{superlattice}$ that exhibits K‐folding. Using scanning tunneling microscopy and spectroscopy (STM/STS), we show that the Γ‐folded system preserves all underlying symmetries, while the K‐folded system exhibits a breaking of the $C_{3}\mathrm{symmetry}$. This symmetry breaking is not electronically driven but originates from variations in the local stacking registry between graphene and NbSe_2_, which modulate the interlayer hybridization.

## 2 Results

Through mechanical stacking, we create near‐commensurate alignments between the $\sqrt{13}\times \;\sqrt{13}\;R13.9^{\circ }\mathrm{CDW}$ in 1T‐NbSe_2_, which itself was created by nano‐phase engineering, and a monolayer of graphene (Figure 1a and Experimental Section 4). The condition for an exact commensurate superlattice is given by the linear Diophantine equation [36, 37]:

(1) $$mR\left(\theta \right)a_{G1}+nR\left(\theta \right)a_{G2}={ja}_{\mathrm{CDW}1}+{ka}_{\mathrm{CDW}2}$$

with $a_{G1}$ and $a_{G2}$ as the primitive lattice vectors of graphene, $a_{\mathrm{CDW}1}$ and $a_{\mathrm{CDW}2}$ as those of the CDW in 1T‐NbSe_2_, and R(θ) as the rotation of the graphene lattice with respect to the NbSe_2_atomic lattice by an angle θ. Since the $\sqrt{13}\times \;\sqrt{13}\;{R13.9}^{\circ }\mathrm{CDW}$ is commensurately locked to the underlying NbSe_2_lattice with an angular offset of 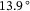, we use the graphene/NbSe_2_atomic‐lattice twist angle θ throughout for convenience (Supporting Information 1). The integers m,n,j, and k define the commensurate condition.

**FIGURE 1:**
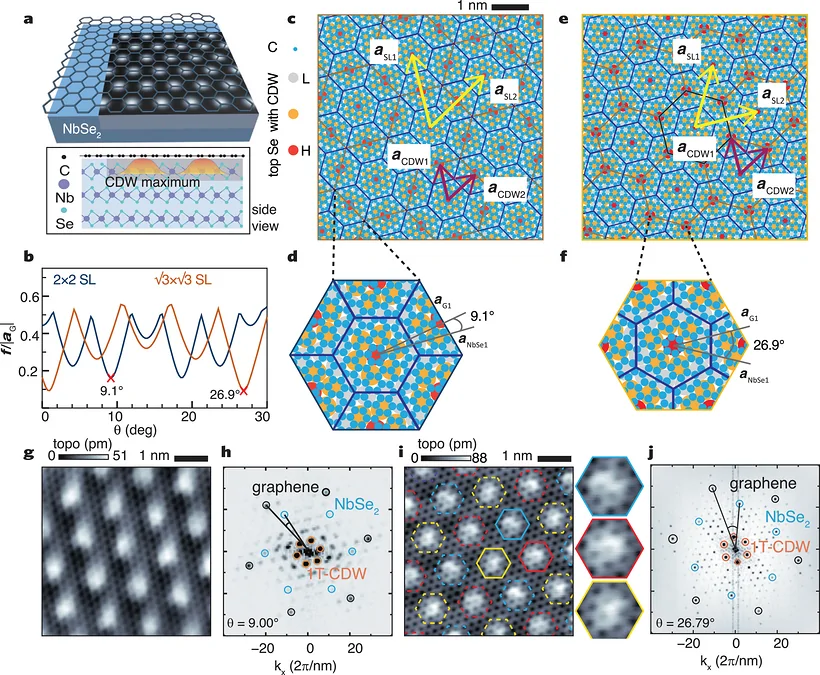
Superlattice engineering. (a) Scheme of the graphene on 1T‐NbSe_2_/2H‐NbSe_2_van der Waals heterostructure. The side view illustrates the charge density wave (CDW) maxima. (b) Twist‐angle θ dependent mismatch between the graphene lattice and the 2×2 (blue) and $\sqrt{3}\times \;\sqrt{3}\;R30^{\circ }$(orange) superlattice of the 1T‐CDW in units of the graphene lattice constant. Here, we use the Wood's notation $\sqrt{3}\times \;\sqrt{3}\;R30^{\circ }\mathrm{to}$ denote a superlattice with unit vectors $\sqrt{3}$ times larger than those of the CDW lattice, rotated by $30^{\circ }$ [38]. (c) Scheme of the stacking configuration of a 2×2commensurate superlattice realized by compressing the graphene lattice by a heterostrain of ε=4.1%. (d) Zoom‐in of the supercell. (e,f) Same as (c,d) but for a $\sqrt{3}\times \;\sqrt{3}\;R30^{\circ }\mathrm{commensurate}$ superlattice with ε=1.8%. (g,h) Atomically resolved STM topography and its FFT with 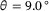 and ε=0.3% (V=0.5V, I=200pA). Here, θ is the twist angle between the graphene and 1T‐NbSe_2_atomic lattices; the corresponding rotation between graphene and the CDW superlattice is thus θ+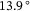. (i,j) Same as (g,h) for the sample with $\theta =26.8^{\circ }$ and ε=0.4% (V=0.3V, I=60pA). Colored hexagons mark the 3 different CDW maxima which repeat periodically, building up the $\sqrt{3}\times \;\sqrt{3}\;R30^{\circ }\mathrm{supercell}$.

However, the different periodicities of the 1T‐NbSe_2_CDW and the graphene lattice mean that Equation (1) has no integer solution. This geometric incommensurability motivates a mismatch function that quantifies how closely the two lattices can approach commensurability:

(2) $$f\left(\theta \right)=\min_{\begin{array}{c} m,n\in Z \end{array}}\left|m\;R\left(\theta \right)\;a_{G1}+n\;R\left(\theta \right)\;a_{G2}-j\;a_{\mathrm{CDW}1}-k\;a_{\mathrm{CDW}2}\right|.$$

This function computes the minimal distance between any rotated graphene lattice vector (spanned by the integers m and n, and the rotation angle θ) and the target CDW superlattice vector. The results for a 2×2superlattice (with j=2 and k=0) and a $\sqrt{3}\times \;\sqrt{3}\;R30^{\circ }\mathrm{superlattice}$ (with j=k=1) are shown in Figure 1b (for additional solutions see Supporting Information 2). Note that, although the experimental twist angle selects which near‐commensurate superlattice geometry is accessible, the subsequent lock‐in of the local registry and residual mismatch is governed by interlayer energetics. These two geometries are of particular interest, because they produce the two distinct band‐folding scenarios: the 2×2superlattice, whose stacking configuration is shown in Figure 1c,d exhibits Γ‐folding, while the $\sqrt{3}\times \;\sqrt{3}\;R30^{\circ }\mathrm{superlattice}$ (stacking configuration in Figure 1e,f) shows K‐folding, preserving valley identity.

Figure 1g–j show atomically resolved STM topographies and the corresponding fast Fourier transformations (FFTs) of two samples with chosen θ close to those for near‐commensurate 2×2and $\sqrt{3}\times \;\sqrt{3}\;R30^{\circ }\mathrm{superlattices}$. Both topographic images reveal a strong periodic height modulation of the graphene layer, imprinted by the underlying triangular CDW lattice of the 1T‐NbSe_2_. The CDW maxima, which correspond to the centers of the Star‐of‐David clusters characteristic of the 1T phase, appear bright in the topography. Following the procedure described in ref. [39], we extract from the FFTs the twist angle θ and a very small heterostrain ε<0.5% in both samples (Supporting Information 3). The deviation by approximately 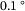 from their ideal commensurate angles has a negligible effect (Supporting Information 4).

Despite their similarities, the topographies of the two samples differ in a revealing way. In the 2×2superlattice, all CDW maxima show a nearly identical local contrast (Figure 1g), whereas three distinct stacking configurations that repeat periodically across the surface can be identified in the $\sqrt{3}\times \;\sqrt{3}\;R30^{\circ }\mathrm{system}$ (Figure 1i). This suggests that in the $\sqrt{3}\times \;\sqrt{3}\;R30^{\circ }\mathrm{superlattice}$, the precise location of the carbon atoms with respect to the CDW lattice has a stronger influence on the interactions between the two layers than in the 2×2superlattice. As we will discuss below, this difference affects the electronic structure and the symmetries of the two samples.

We begin by examining the 2×2superlattice using differential conductance (dI/dV) spectroscopy, whose intensity is proportional to the local density of states (LDOS). Figure 2a shows dI/dVspectra acquired along a line, starting at the center of the CDW maximum (referred to as A‐site), and moving toward the midpoint between two neighboring clusters (B‐site). At the A‐site, the dI/dVspectrum exhibits three pronounced peaks at energies $E_{L1}$, $E_{L2}$, and $E_{L3}$ (Supporting Information 5 and 6). These peaks are absent in measurements taken at 1T/2H‐NbSe_2_or graphene/2H‐NbSe_2_heterostructures (Figure 2b), confirming that they arise specifically from the interaction between the graphene sheet and the 1T‐NbSe_2_layer.

**FIGURE 2:**
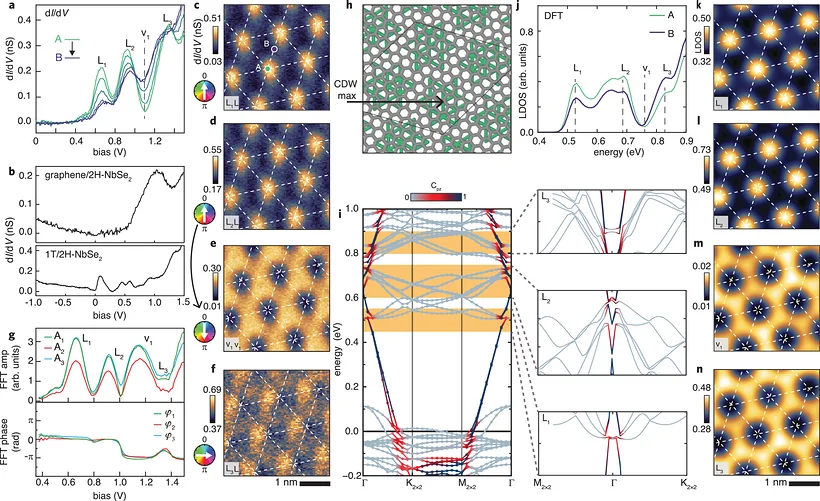
Electronic structure of the 2×2 superlattice. (a) Spectra measured between the A‐ and B‐sites, marked in panel c, show three peaks at energies $E_{L1}=0.66\left(1\right)\;\mathrm{eV}$, $E_{L2}=0.92\left(1\right)\;\mathrm{eV}$, and $E_{L3}=1.36\left(1\right)\;\mathrm{eV}$ and a dip at $E_{v1}=1.10\left(1\right)\;\mathrm{eV}$. (b) On graphene/2H‐NbSe_2_and 1T‐NbSe_2_/2H‐NbSe_2_these features are absent. (c–f) dI/dVmaps measured at biases corresponding to $E_{L1-L3}$ and $E_{v1}$. (g) Evolution of the FFT amplitudes $A_{1-3}$ (top) and phases $\varphi_{1-3}$ (bottom) extracted from the dI/dVmaps. (h) Schematic model used for the DFT calculations. (i) Calculated band structure of the superlattice mBZ and zoom‐ins to the bands near the $L_{1-3}$ states. The hybridized states are coded in red. (j) Calculated LDOS at the A‐ and B‐sites. (k–n) Calculated LDOS maps at energies corresponding to the $L_{1-3}$ and $v_{1}$ features.

As the tip moves from the A‐ to the B‐site, the peaks decay in intensity, while a new feature at $E_{v1}$ emerges with maximum intensity at the B‐site. These complementary behaviors are visualized directly in constant energy dI/dVmaps. At $E_{L1}$ and $E_{L2}$, the dI/dVmaps exhibit a dot‐like structure with high intensity at the A‐sites (Figure 2c,d). The FFTs of these maps reveal significant intensity exclusively at the Bragg peaks corresponding to the reciprocal vectors of the CDW and $Q_{i}^{\text{CDW}}$, suggesting that the spatial variation of the dI/dVcan be described by a Fourier series expansion over these wavevectors as:

(3) $$dI/dV\left(r,V\right)=A_{0}\left(V\right)+\sum_{i=1}^{3}A_{i}\left(V\right)\;\cos\;\left(Q_{i}^{\mathrm{CDW}}\;\cdot \;r+\varphi_{i}\left(V\right)\right).$$

In this equation, $A_{1,2,3}$ are the amplitudes and $\varphi_{1,2,3}$ are the phase shifts of the LDOS modulation along the three fundamental directions of the CDW [40] (see Supporting Information 7). The phases $\varphi_{i}\left(V\right)$ define the real‐space registry of the LDOS modulation relative to the CDW superlattice. When $\varphi_{i}=0$, the dI/dVmaxima align with the A‐sites, producing the dot‐like pattern as seen at $L_{1}$ and $L_{2}$. Near V≈1V, all three phases simultaneously undergo a π‐shift, resulting in an inversion of the LDOS contrast and a transformation of the dot‐like pattern into a honeycomb‐like structure (Figure 2e). At the energy of $L_{3}$, the phases adopt intermediate values, $\varphi_{1-3}\approx -\pi /2$, shifting the intensity maxima to positions between the A‐ and B‐sites (Figure 2f). A significant finding of this analysis is that $A_{1-3}$ and $\varphi_{1-3}$ evolve similarly across the measured energy range (Figure 2g), indicating the preservation of the underlying $C_{3}$ rotational symmetry of the CDW lattice. The small difference in relative intensity might originate from a slight directional dependence of the probing tip.

To understand the origin of the observed spectral features, we performed DFT calculations (see Experimental Section 4) for a commensurate 2×2superlattice (Figure 2h). This commensurate superlattice is achieved by introducing an isotropic strain to the graphene sheet to absorb the mismatch, while the NbSe_2_lattice and the CDW superlattice are kept at their relaxed lattice constants. This isotropic strain does not introduce a directional bias for the symmetry analysis. The superlattice potential defines the mBZ into which the original bands are folded (Figure 2i). The key feature of this geometry is the Γ‐folding: the Dirac cones from both the K and $K^{\prime }$ valleys of pristine graphene folds onto the Γ point of the mBZ. This folding follows directly from the designed superlattice geometry: graphene K and $K^{\prime }$ coincide with superlattice reciprocal vectors and therefore fold to Γ in the reduced mBZ (Supporting Information 8). This valley degeneracy, combined with hybridization with the NbSe_2_bands, produces peaks in the calculated LDOS (Figure 2j) and the corresponding LDOS maps (Figure 2k–n), which qualitatively reproduce the experimental observations.

We now turn to the electronic structure of the $\sqrt{3}\times \;\sqrt{3}\;R30^{\circ }\mathrm{superlattice}$. At the A‐site, we observe spectral features that are similar to those in the 2×2superlattice, albeit with a subtle shift of the peak positions (Figure 3a). The three B‐sites, which are related by a 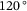 rotation around the A‐site, however, are electronically inequivalent: The $B_{1}$‐site shows an additional peak ($L_{1}^{\prime }$) and increased intensity at $L_{1}$, but decreased intensity at $L_{2}$, compared to the $B_{2}$‐ and $B_{3}$‐sites (Figure 3b). Applying the same FFT phase analysis to this superlattice reveals a much more complex behavior (Figure 3c): At the energy of peak $L_{1}$, we observe a dot‐like pattern, accompanied by stripes along one direction (Figure 3d), resulting in amplitudes $A_{1}\approx A_{3}\ll A_{2}$ and nearly aligned phases $\left(\varphi_{1-3}\approx 0\right)$, providing a clear evidence of broken $C_{3}\mathrm{symmetry}$. As the energy increases to $L_{2}$, the $C_{3}\mathrm{symmetry}$ is restored, yielding a lattice of symmetric circular dots at A‐sites (Figure 3e).

**FIGURE 3:**
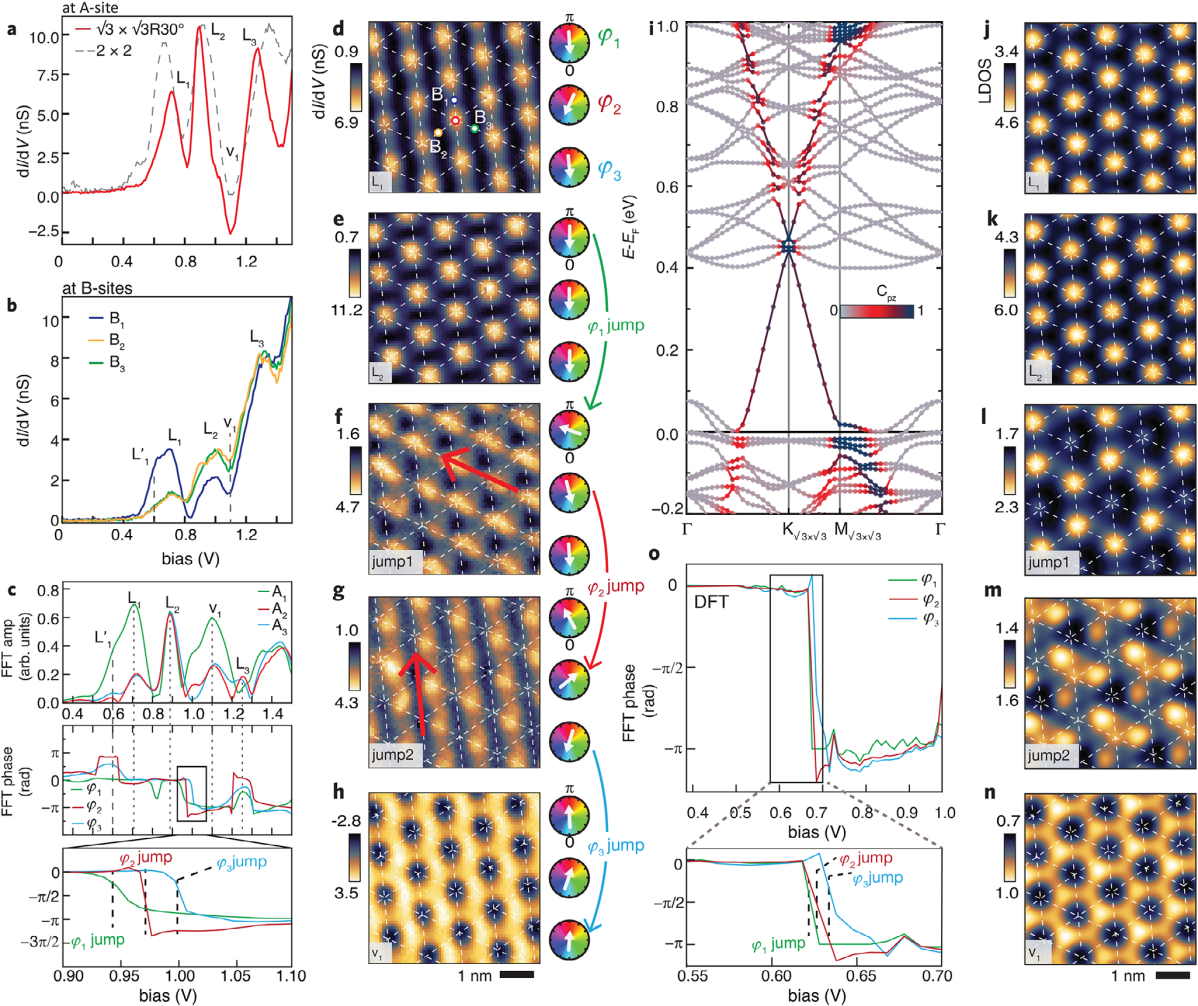
Electronic structure of the $\sqrt{3}\times \;\sqrt{3}\;R30^{\circ }$ superlattice. (a) Spectrum taken at the A‐site (solid line) shows peaks at energies $E_{L1}=0.71\pm 0.01$ eV, $E_{L2}=0.90\pm 0.01$ eV, and $E_{L3}=1.27\pm 0.01$ eV and a dip at $E_{v1}=1.10\pm 0.01$ eV. The spectrum measured in the 2×2 superlattice (dashed line, ×25) is shown for comparison. (b) Spectra recorded at the CDW minima $B_{1}$ (blue), $B_{2}$ (orange), and $B_{3}$ (green), which are rotated around the CDW maximum by 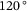 with respect to each other. At $B_{1}$ an additional peak $L_{1}^{\prime }$ appears at $E_{L1^{\prime }}=0.62\pm 0.01$ eV. (c) Phase and amplitude from the FFT analysis of the dI/dVdata as a function of bias. (d–h) dI/dVmaps at the indicated energies. (i) Calculated band structure of the $\sqrt{3}\times \;\sqrt{3}\;R30^{\circ }\mathrm{superlattice}$ mini‐Brillouin zone. (j–n) DFT‐calculated LDOS maps, at the corresponding states. (o) Similar phase analysis using the DFT‐calculated LDOS data.

Between $L_{2}$ and $v_{1}$, we again observe a transition from a dot‐like to a honeycomb‐like LDOS pattern, but, unlike the symmetry‐preserving transition in the 2×2system, this transition proceeds through a series of staggered phase jumps that progressively break $C_{3}\mathrm{symmetry}$ (Figure 3c). The first phase jump distorts the dots into ellipses along one axis (Figure 3f); the second phase jump creates a transient stripe pattern (Figure 3g); and the third phase jump completes the transformation to the honeycomb pattern (Figure 3h). However, even at $v_{1}$, the amplitudes remain unequal, with $A_{2}$ being dominant, confirming the persistence of the broken symmetry. Note that this symmetry breaking cannot be induced by an unknown tip geometry (Supporting Information 9).

DFT calculations on this supercell confirm the K‐folding (Figure 3i). Hybridization with the 1T‐NbSe_2_bands gives rise to a series of prominent peaks, and the calculated LDOS maps qualitatively reproduce the measured dI/dVmaps (Figure 3j–n). Notably, the same FFT phase analysis applied to the calculated maps reveals a comparable series of staggered phase jumps (Figure 3o), yielding asymmetric intermediate states with broken $C_{3}\mathrm{symmetry}$ (Figure 3m). Here, the calculated and measured transient textures nevertheless differ (e.g., Figure 3l,f), because the real‐space pattern also depends on the amplitudes of the three CDW components, which are strongly unequal in the experiment but remain nearly equal in the calculation

Although the DFT calculations capture the broken symmetry in the $\sqrt{3}\times \;\sqrt{3}\;R30^{\circ }\mathrm{superlattice}$ and its absence in the 2×2superlattice, the microscopic origin of this symmetry breaking remains unclear within DFT. To elucidate the mechanism, we developed a simplified atomistic model derived from the microscopic interlayer tunneling model that quantifies the spatial average of the interlayer coupling based on local atomic registry. This model enables us to simulate the influence of lateral sliding of the graphene layer with respect to the NbSe_2_and the influence of isotropic strain on the interlayer hybridization.

Our model quantifies the net directional preference of hybridization between the $p_{z}$ orbitals of the carbon atoms in the graphene layer and the p orbitals of the topmost Se atoms in the NbSe_2_layer (Figure 4a):

(4) $$d_{pp}=\sum_{i,j}\frac{r_{C,i}-r_{\mathrm{Se},j}}{\left|r_{C,i}-r_{\mathrm{Se},j}\right|}w\;\left(\left|r_{C,i}-r_{\mathrm{Se},j}\right|\right)\;,$$

where the sum runs over all C atoms of the graphene, and all Se atoms in the topmost NbSe_2_layer, and $r_{C,i}$ ($r_{\mathrm{Se},j}$) denotes the position of the i th carbon (j th selenium) atom. The exponential weighting function $w\left(r\right)=e^{-\alpha r}$ represents the distance dependence of Slater–Koster tight‐binding parameters arising from orbital overlap [41]. The decay constant α=0.3 Å^−1^ ensures that nearest‐neighbor C–Se pairs dominate the hybridization. Further details are provided in Supporting Information 10.

**FIGURE 4:**
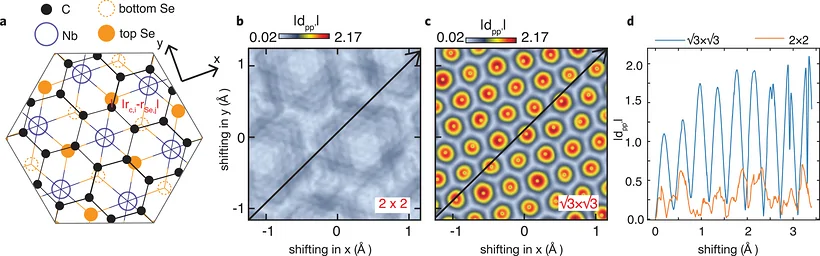
Geometric origin of the symmetry breaking. (a) Schematic of the interlayer interaction model. (b) 2D resolved sliding map of the calculated hybridization magnitude $|d_{pp}|$ for the 2×2superlattice with sliding directions defined in (a). (c) 2D resolved sliding map of the $|d_{pp}|$ for the $\sqrt{3}\times \;\sqrt{3}\;R30^{\circ }\mathrm{superlattice}$. (d) Comparison of the $|d_{pp}|$ as a function of sliding between these two commensurate configurations, along the sliding paths indicated in (b and c).

The results for laterally sliding graphene in the commensurate 2×2and $\sqrt{3}\times \;\sqrt{3}\;R30^{\circ }\mathrm{superlattices}$ are shown in Figure 4b–d, and reveal a striking difference. In the 2×2superlattice, $d_{pp}$ remains nearly constant under sliding, indicating a flat hybridization landscape that is insensitive to the local registry. In the $\sqrt{3}\times \;\sqrt{3}\;R30^{\circ }\mathrm{superlattice}$, by contrast, the average hybridization is strongly modulated.

This different response enables us to address the origin of the symmetry breaking. While band folding only fixes the appearance of the graphene Dirac cones in the mBZ, it cannot by itself lower the point‐group symmetry: both superlattices are $C_{3}$‐symmetric, and K‐folding simply permutes the mBZ corners under a 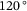 rotation. The observed symmetry breaking must therefore come from real‐space relaxation. In the $\sqrt{3}\times \;\sqrt{3}\;R30^{\circ }\mathrm{geometry}$, the hybridization landscape of the ideal structure is highly susceptible to perturbations; even a small lateral shift or strain can break the delicate balance of the three equivalent CDW directions and lift their degeneracy. The 2×2geometry, with its smoother hybridization landscape, is more robust against such perturbations and maintains $C_{3}\mathrm{symmetry}$ even under realistic sample conditions. Therefore, the symmetry breaking observed in the $\sqrt{3}\times \;\sqrt{3}\;R30^{\circ }\mathrm{superlattice}$ arises from a geometric instability that is intrinsic to this superlattice configuration. Consistently, the sliding map of $d_{pp}$ is itself $C_{3}$‐symmetric (Figure 4b), so the model imposes no directional bias. The same contrast persists for genuinely incommensurate lattices (Supporting Information 10), showing that the $C_{3}$‐breaking propensity is intrinsic to the registry landscape of the $\sqrt{3}\times \;\sqrt{3}\;R30^{\circ }\mathrm{geometry}$ rather than an artefact of enforced commensuration.

## 3 Conclusion

The superlattice engineering strategy demonstrated here can be naturally extended to a broad class of vdW heterostructures beyond our specific example.

By selecting host materials with different CDW periodicities and symmetries, one can systematically design band‐folding pathways and target specific points of the mBZ for Dirac cones or other band features. Promising candidates include graphene on 1T‐${\mathrm{TaS}}_{2}$ [21], whose $\sqrt{13}\times \;\sqrt{13}\;R13.9^{\circ }\mathrm{CDW}$ has a slightly larger periodicity than that of 1T‐${\mathrm{NbSe}}_{2}$, as well as other transition‐metal dichalcogenides with commensurate or nearly commensurate CDWs. In such systems, the competition between different registries, coupled to the structural instabilities revealed in this work, is expected to generate a rich landscape of symmetry‐broken ground states, including nematic and ferroic‐like orders that could be tuned by strain, pressure, or electrostatic gating.

Our approach can be extended to semiconducting 2D crystals such as ${\mathrm{WSe}}_{2}$ on 1T‐NbSe_2_or 1T‐${\mathrm{TaS}}_{2}$. In these heterostructures, CDW‐induced superlattices provide a route to engineer not only electronic minibands but also excitonic dispersions, valley selection rules, and radiative lifetimes through zone folding of exciton states [42]. This opens opportunities to realize designer excitonic lattices, control interlayer, and moiré‐like excitons [43]. More broadly, combining CDW‐based proximity superlattices with twist, strain, or multilayer stacking could enable hybrid CDW‐moiré platforms in which structural relaxation, electronic correlations, and topology are all on comparable energy scales. Our results thus establish CDW‐mediated superlattice engineering as a robust and deterministic strategy for creating and controlling emergent quantum phases in vdW heterostructures, and motivate future experiments that exploit structural degrees of freedom as an equal partner to electronic interactions in the design of quantum materials.

## 4 Experimental Section 

### 4.1 Sample Fabrication

We fabricated the heterostructures in an Ar‐filled glove box following a suspended dry pickup and flip‐over assembly technique [44, 45]. During assembly, the graphene and NbSe_2_crystallographic edges were aligned optically under the transfer microscope to the targeted orientation [46]. We then placed the assembled heterostructure on top of a 285nm Si/${\mathrm{SiO}}_{2}$ chip on which we constructed contact and tip guidance electrodes [47] by electron beam lithography (Supporting Information 11).

Initial STM topographies of the graphene/2H‐NbSe_2_heterostructures resolve the graphene lattice, the underlying NbSe_2_lattice, and the resulting moiré superlattice, but show no signatures of the 3×3 CDW order (Supporting Information 12), consistent with previous reports [48]. To realize graphene on 1T‐NbSe_2_, we employ local bias pulsing with the STM tip on graphene/2H‐NbSe_2_ [49], which induces a controlled phase transition from the 2H to the 1T polytype in the NbSe_2_layer beneath the graphene (Supporting Information 13).

### 4.2 STM and STS Measurements

All measurements were performed in ultra‐high vacuum ($\leq 1\times 10^{-10}\;\mathrm{mbar}$) and at a base temperature of T=6K. We used electrochemically etched tungsten tips, flashed in ultra‐high vacuum to remove the oxide layer and contamination.

Additionally, the tip was prepared in situ by repeatedly indenting it into the deposited gold contacts until it showed a flat density of states. STS was performed using a standard lock‐in technique at a modulation frequency of 877Hz and modulation voltage $V_{\mathrm{mod}}=10$ mV.

### 4.3 DFT Calculations

Density functional theory calculations were performed in a plane‐wave pseudopotential framework. We first relaxed the $\sqrt{13}\times \;\sqrt{13}\;R13.9^{\circ }$ CDW unit cell of 1T‐NbSe_2_with fixed in‐plane lattice parameters, and then constructed commensurate 2×2 and $\sqrt{3}\times \;\sqrt{3}\;R30^{\circ }$ graphene/1T‐NbSe_2_supercells by straining graphene homoaxially and twisting its lattice vector. The supercells are those shown in Figure 1d,f; they are the minimal commensurate cells that contain all local registries between graphene and the CDW of 1T‐NbSe_2_, namely, 2×2and $\sqrt{3}\times \;\sqrt{3}\;R30^{\circ }\mathrm{superlattice}$. The graphene layer was initially placed ∼5 Å above the Nb plane, and all atomic positions in the heterostructures were relaxed. We used a generalized‐gradient exchange–correlation functional with vdW corrections, ultrasoft pseudopotentials, a vacuum spacing of 20 Å, and kinetic‐energy cutoffs of 40/400 Ry for wavefunctions/charge density. Moderately dense k‐point meshes were employed for self‐consistent calculations and further refined for non‐self‐consistent band‐structure, (projected) DOS and real‐space LDOS calculations. The resulting electronic structures capture the key qualitative differences between the 2×2 and $\sqrt{3}\times \;\sqrt{3}\;R30^{\circ }$ superlattices, including their distinct band folding and symmetry properties. More details can be found in the Supporting Information 14.

## Author Contributions

K. J.,  J. M.‐C, and M. T. conceived the project. K. J. fabricated the samples. K. J., J. Z., and J. M.‐C. performed the STM experiments. K. J. and M. T. analyzed the data. L. K., Z. A. H. G., and D. M. K. performed the DFT and the geometry model calculations. F. L., D. M. K., J. M.‐C., and M. T. supervised the project. K. J. and M. T. wrote the manuscript. All authors discussed and commented on the manuscript.

## Conflicts of Interest

The authors declare no conflicts of interest.

## Supporting information


**Supporting Information**: adma74569‐sup‐0001‐SuppMat.pdf.

## Data Availability

All data presented in this study are available on the Jülich Data Repository at https://doi.org/10.26165/JUELICH‐DATA/H8154J.
