# Supplementary material for: Tailoring Symmetry Breaking in Engineered van der Waals Superlattices
Source: Adv Mater. 2026 Aug 11;38(57):e74569. doi: 10.1002/adma.74569 (PMC13660823; doi:10.1002/adma.74569)
Supplement: Supplementary file 1 [file ADMA-38-e74569-s001.pdf]

Supporting Information for
Tailoring symmetry breaking in engineered van der Waals superlattices

Keda Jin[1,2,3], Lennart Klebl[4], Zachary A. H. Goodwin[5,6], Junting Zhao[1,2,3], Felix Lüpke[1,2,7], Dante M. Kennes[8,2,9], Jose Martinez-Castro[1,2,3], Markus Ternes[1,2,3]

[1]Peter Grünberg Institut (PGI-3), Forschungszentrum Jülich, 52425 Jülich, Germany

[2]Jülich Aachen Research Alliance, Fundamentals of Future Information Technology, 52425 Jülich, Germany

[3]Institut für Experimentalphysik II B, RWTH Aachen, 52074 Aachen, Germany

[4]Institut für Theoretische Physik und Astrophysik and Würzburg-Dresden Cluster of Excellence ct.qmat, Universität Würzburg, 97074 Würzburg, Germany

[5]Department of Materials, University of Oxford, Parks Road, Oxford OX1 3PH, United Kingdom

[6]John A Paulson School of Engineering and Applied Sciences, Harvard University, Cambridge, MA, USA

[7]Institute of Physics II, Universität zu Köln, Zülpicher Straße 77, 50937 Köln, Germany

[8]Institut für Theorie der statistischen Physik, RWTH Aachen, 52074 Aachen, Germany

[9]Max Planck Institute for the Structure and Dynamics of Matter, Center for Free Electron Laser Science, 22761 Hamburg, Germany

CONTENTS

S1. Relation between the graphene/$NbSe_2$ atomic moiré and the CDW superlattice 4

S2. Additional almost commensurate superlattices 5

S3. Determination of the twist angle and heterostrain in the graphene layer 7
A. Extract heterostrain 7
B. Local relaxation across the 2H/1T phase boundary 9

S4. Tolerance of the folding configurations to twist-angle deviations 11
A. Sensitivity of the mismatch function 11
B. Robustness of the Γ/K folding against the rotational mismatch 12
C. Impact on the local stacking registry 14

S5. Wide range STS spectroscopy 15

S6. Determination of the peak positions 16

S7. Extraction of the form factor 17
A. 1D illustration 17
B. Extension to 2D triple-q CDW 17
C. Extraction of the form factor from the calculated LDOS maps 21

S8. Band-folding analysis of the 2×2 and $\sqrt{3}\times\sqrt{3}\,\mathrm{R}30^\circ$ superlattices 22

S9. Exclusion of a tip-induced origin of the symmetry breaking 24
A. The symmetry-breaking observables are not due to tip directionality 24
B. Bias-dependent topography 25
C. Single-frame comparison across a phase boundary 26

S10. Geometric model of interlayer hybridisation anisotropy 27
A. Connection to microscopic interlayer tunnelling 27
B. Orbital selectivity 28
C. Sensitivity to the decay constant α 28
D. Symmetry of the sliding map and its relation to C_3 breaking 29

S11. Optical images of the device 32

S12. Topography of the two samples before the nanoscale phase engineering 33

S13. Nanoscale Engineering to generate graphene/1T-$NbSe_2$ samples 34

S14. Details of the DFT calculations 35

References 37

S1. RELATION BETWEEN THE GRAPHENE/NBSE$_2$ ATOMIC MOIRÉ AND THE CDW SUPERLATTICE

In this section, we show that the atomic moiré arising between graphene and the $NbSe_2$ atomic lattice is also commensurate to the moiré between graphene and the CDW superlattice. The first graphene/$NbSe_2$ atomic moiré [1] has a short periodicity ($\sim 0.48\,\mathrm{nm}$, see Sec. S3), well below the CDW periodicity ($|\boldsymbol{a}_{\mathrm{CDW}}| = 1.24\,\mathrm{nm}$), so the two appear at clearly separated wavevectors in reciprocal space.

To find out the high order moiré between graphene and $NbSe_2$, we follow the equation 1 and equation 2 in the main text, where the commensurate condition between the rotated graphene lattice and the $NbSe_2$ is defined by:

$$m\mathbf{R}(\theta)\boldsymbol{a}_{\mathrm{G1}} + n\mathbf{R}(\theta)\boldsymbol{a}_{\mathrm{G2}} = j'\boldsymbol{a}_{\mathrm{NbSe1}} + k'\boldsymbol{a}_{\mathrm{NbSe2}}. \tag{S1.1}$$

To find the moire periodicity, we need to minimize this function:

$$f(\theta) = \min_{m,n\in\mathbb{Z}} \left| m\,\mathbf{R}(\theta)\,\boldsymbol{a}_{\mathrm{G1}} + n\,\mathbf{R}(\theta)\,\boldsymbol{a}_{\mathrm{G2}} - j'\,\boldsymbol{a}_{\mathrm{NbSe1}} - k'\,\boldsymbol{a}_{\mathrm{NbSe2}} \right|. \tag{S1.2}$$

Since the CDW is a commensurate $\sqrt{13}\times\sqrt{13}$ reconstruction of the $NbSe_2$ atomic lattice, the atomic lattice vectors can be written in terms of the CDW lattice vectors as:

$$\begin{pmatrix} a_{\mathrm{NbSe1}} \\ a_{\mathrm{NbSe2}} \end{pmatrix} = \frac{1}{13}\begin{pmatrix} 4 & -1 \\ 1 & 3 \end{pmatrix}\begin{pmatrix} a_{\mathrm{CDW1}} \\ a_{\mathrm{CDW2}} \end{pmatrix} \tag{S1.3}$$

so that we can rewrite the minimization of Eq. (S1.2) entirely in terms of the CDW lattice vectors.

$$f(\theta) = \min_{m,n\in\mathbb{Z}} \left| m\,\mathbf{R}(\theta)\,\boldsymbol{a}_{\mathrm{G1}} + n\,\mathbf{R}(\theta)\,\boldsymbol{a}_{\mathrm{G2}} - \frac{1}{13}\left[(4j'+k')\,\boldsymbol{a}_{\mathrm{CDW1}} + (3k'-j')\,\boldsymbol{a}_{\mathrm{CDW2}}\right] \right|. \tag{S1.4}$$

When $j' = 6$ and $k' = 2$, the bracketed term in Eq. (S1.4) reduces to $2\,\boldsymbol{a}_{\mathrm{CDW1}}$, i.e. the 2×2 superlattice discussed in the main text; and when $j' = 2$ and $k' = 5$, it reduces to $\boldsymbol{a}_{\mathrm{CDW1}} + \boldsymbol{a}_{\mathrm{CDW2}}$, i.e. the $\sqrt{3}\times\sqrt{3}\,\mathrm{R}30^\circ$ superlattice discussed in the main text. This means that the moiré between the graphene and the CDW superlattice we discussed in the main text is also the moiré between the graphene and the $NbSe_2$ atomic lattice with a higher-order commensuration. Consequently, the graphene/$NbSe_2$ atomic moiré does not introduce an independent periodicity at the analysed length scale: it is commensurate with, and folds into, the same CDW superlattice whose form factors we extract.

S2. ADDITIONAL ALMOST COMMENSURATE SUPERLATTICES

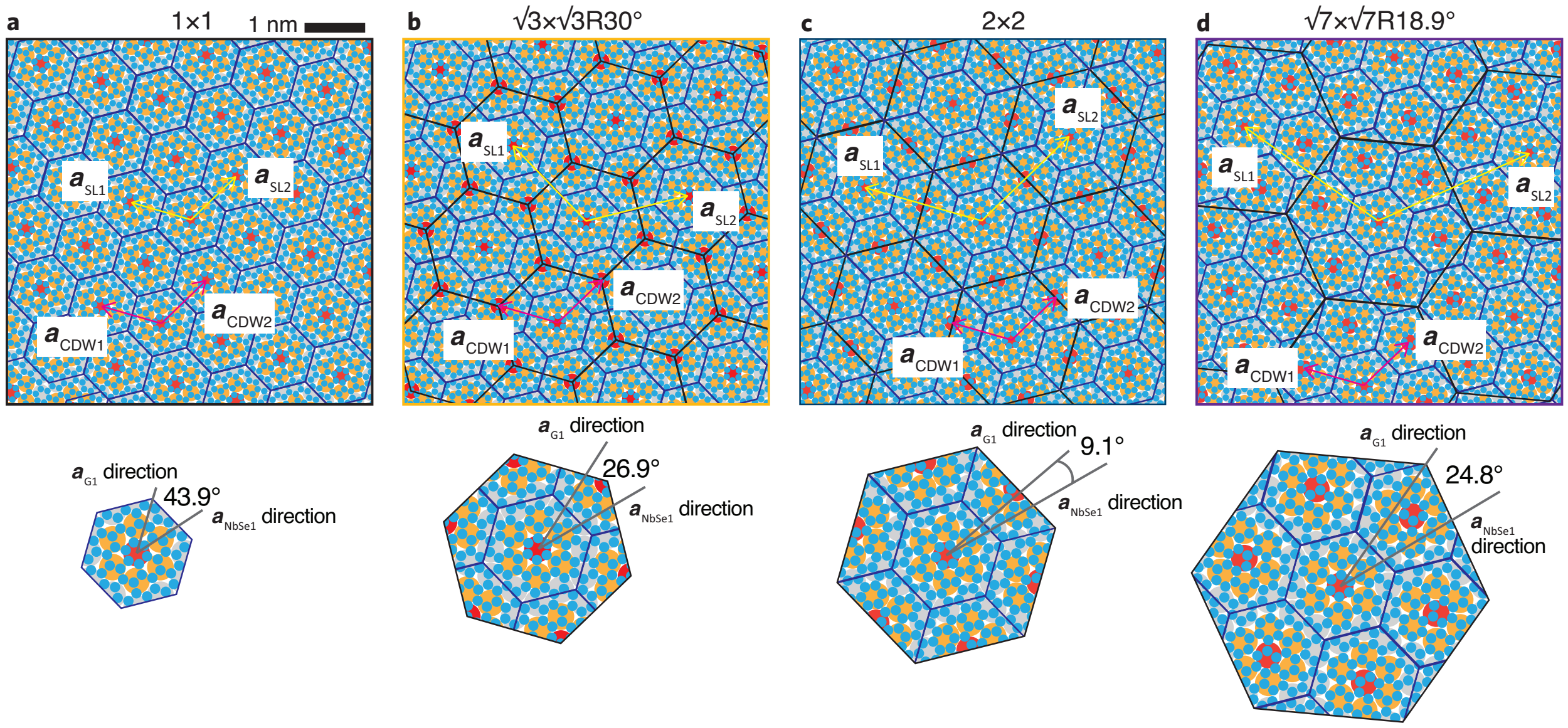

FIG. S2.1. **Real-space models of almost commensurate graphene/1T-NbSe$_2$ heterostructures.** Schematic top views of four twist angles that produce superlattices with **a,** 1×1, **b,** $\sqrt{3} \times \sqrt{3}$ R30°, **c,** 2×2, **d,** $\sqrt{7} \times \sqrt{7}$ R19.1° periodicity. Yellow arrows mark the superlattice primitive vectors $\boldsymbol{a}_{\mathrm{SL1,\,SL2}}$; magenta arrows indicate the primary CDW vectors $\boldsymbol{a}_{\mathrm{CDW1,\,CDW2}}$ of 1T-NbSe$_2$. Only the C atoms of the graphene lattice (blue) and the Nb atoms of NbSe$_2$ (gray, orange, and red, color codes the CDW modulation) drawn for clarity. Insets beneath each panel depict the relative orientation between a graphene lattice vector $\mathbf{a}_{\mathrm{G1}}$ and NbSe$_2$ lattice vector $\mathbf{a}_{\mathrm{NbSe1}}$ together with the corresponding misalignment angle.

The commensurate condition between the rotated graphene lattice and the CDW superlattice is defined by equation (1) of the main text:

$$m\mathbf{R}(\theta)\boldsymbol{a}_{\mathrm{G1}} + n\mathbf{R}(\theta)\boldsymbol{a}_{\mathrm{G2}} = j\boldsymbol{a}_{\mathrm{CDW1}} + k\boldsymbol{a}_{\mathrm{CDW2}}, \tag{S2.1}$$

where (j, k) are integers that specify the target superlattice on the CDW lattice. For the hexagonal CDW lattice with primitive vectors $\boldsymbol{a}_{\mathrm{CDW1}}$ and $\boldsymbol{a}_{\mathrm{CDW2}}$ separated by an angle of $60°$, the superlattice vector $\boldsymbol{a}_{\mathrm{SL}} = j\,\boldsymbol{a}_{\mathrm{CDW1}} + k\,\boldsymbol{a}_{\mathrm{CDW2}}$ has a length of:

$$|\boldsymbol{a}_{\mathrm{SL}}| = |\boldsymbol{a}_{\mathrm{CDW}}|\,\sqrt{j^2 + k^2 + jk} \tag{S2.2}$$

and is rotated relative to $\boldsymbol{a}_{\mathrm{CDW1}}$ by an angle of

$$\phi = \arccos\left(\frac{2j + k}{2\sqrt{j^2 + jk + k^2}}\right). \tag{S2.3}$$

TABLE S2.1. Superlattice configurations for different (j, k) values.

$(j,\, k)$	p	ϕ (°)	Wood's notation
(1, 0)	1	0	1×1
(1, 1)	$\sqrt{3}$	30.0	$\sqrt{3} \times \sqrt{3}$ R30°
(2, 0)	2	0	2×2
(2, 1)	$\sqrt{7}$	19.1	$\sqrt{7} \times \sqrt{7}$ R19.1°
(3, 0)	3	0	3×3
(2, 2)	$2\sqrt{3}$	30.0	$2\sqrt{3} \times 2\sqrt{3}$ R30°
(3, 1)	$\sqrt{13}$	13.9	$\sqrt{13} \times \sqrt{13}$ R13.9°

Different choices of (j, k) generate superlattices with distinct periodicities and orientations relative to the underlying CDW lattice. In Wood's notation [2], the resulting superlattice is denoted $p \times p$ Rϕ, where $p = |\boldsymbol{a}_{\mathrm{SL}}|/|\boldsymbol{a}_{\mathrm{CDW}}| = \sqrt{j^2 + k^2 + jk}$. Table S2.1 summarises some lower-order configurations. In the main text, we focus on the $\sqrt{3} \times \sqrt{3}$ R30° and 2×2 superlattices, which realise the two fundamental Dirac-cone folding pathways (K-folding and Γ-folding, respectively). Figure S2.1 broadens this scope by presenting the full set of low-order almost commensurate stackings accessible through twist-angle control.

The 1×1 configuration ($\theta \approx 43.9°$, Supplementary Fig. S2.1a) is the simplest commensurate registry in which the superlattice period coincides with the CDW period itself. In this geometry, the graphene layer experiences the full, unmodified CDW potential without additional spatial averaging, making it a natural reference for isolating the bare proximity effect of the 1T-$NbSe_2$ CDW on graphene's electronic structure.

The $\sqrt{7} \times \sqrt{7}$ R19.1° configuration ($\theta \approx 24.8°$, Supplementary Fig. S2.1d) produces a supercell containing seven CDW unit cells. This results in the smallest mini-Brillouin zone among the four geometries shown. The enlarged real-space periodicity increases the number of folded bands within the mBZ, which could enhance hybridisation between graphene and $NbSe_2$ states and promote the formation of flat bands near the Dirac point. Together, these four configurations span the accessible low-order commensurate conditions of the graphene/1T-$NbSe_2$ system and represent promising targets for future experimental and theoretical investigation.

S3. DETERMINATION OF THE TWIST ANGLE AND HETEROSTRAIN IN THE GRAPHENE LAYER

A. Extract heterostrain

In Gr/1T-$NbSe_2$ samples, strain primarily originates from two sources: the sample fabrication process and the application of a bias pulse by the STM tip. The strain introduced during the sample fabrication, including exfoliation and polymer-based transfer, affects the entire sample uniformly, while voltage pulsing introduces strain locally in proximity to the probing tip. Consequently, for the graphene/2H-$NbSe_2$ region, only the fabrication-induced component is present, whereas the Gr/1T-$NbSe_2$ samples experience both fabrication-induced strain and bias-pulsing strain. To investigate these effects, we quantitatively compare the heterostrain in representative Gr/2H-$NbSe_2$ and Gr/1T-$NbSe_2$.

Our analysis follows the established protocol in Refs. [3, 4]. We begin with the *unstrained* lattices of graphene and $NbSe_2$, each of which is hexagonal and possesses C_3 rotational symmetry. The three symmetry–related reciprocal vectors of graphene are

$$\boldsymbol{k}_{\mathrm{G}}^{(\ell)} = k_{\mathrm{G}}\,\mathbf{R}\big(\tfrac{2\pi}{3}(\ell-1)\big)\,\hat{\boldsymbol{x}}, \qquad \ell = 1,2,3, \tag{S3.1}$$

where $k_{\mathrm{G}} = 4\pi/(\sqrt{3}\,a_{\mathrm{G}})$, $\mathbf{R}(\phi)$ is the rotation matrix:

$$\mathbf{R}(\phi) = \begin{pmatrix} \cos\phi & -\sin\phi \\ \sin\phi & \cos\phi \end{pmatrix},$$

and $\hat{\boldsymbol{x}}$ is the unity vector in x-direction. If the two layers are rotated by the twist angle θ_{t} and differ in lattice constant by the ratio

$$r = \frac{a_{\mathrm{G}}}{a_{\mathrm{NbSe}}},$$

then we can represent the $NbSe_2$ reciprocal vectors by those of the graphene via:

$$\boldsymbol{k}_{\mathrm{NbSe}}^{(\ell)} = r\,\mathbf{R}(\theta_{\mathrm{t}})\,\boldsymbol{k}_{\mathrm{G}}^{(\ell)}, \qquad \ell = 1,2,3. \tag{S3.2}$$

The moiré pattern arises from the difference between the reciprocal vectors of the two constituent lattices. In the absence of strain (Fig. S3.1a), the moiré wavevector $\boldsymbol{k}_{\mathrm{M}}^{(\ell)}$ is simply

$$\boldsymbol{k}_{\mathrm{M}}^{(\ell)} = \boldsymbol{k}_{\mathrm{NbSe}}^{(\ell)} - \boldsymbol{k}_{\mathrm{G}}^{(\ell)}, \tag{S3.3}$$

where $\boldsymbol{k}_{\mathrm{G}}^{(\ell)}$ ($\boldsymbol{k}_{\mathrm{NbSe}}^{(\ell)}$) is the reciprocal vector of graphene ($NbSe_2$).

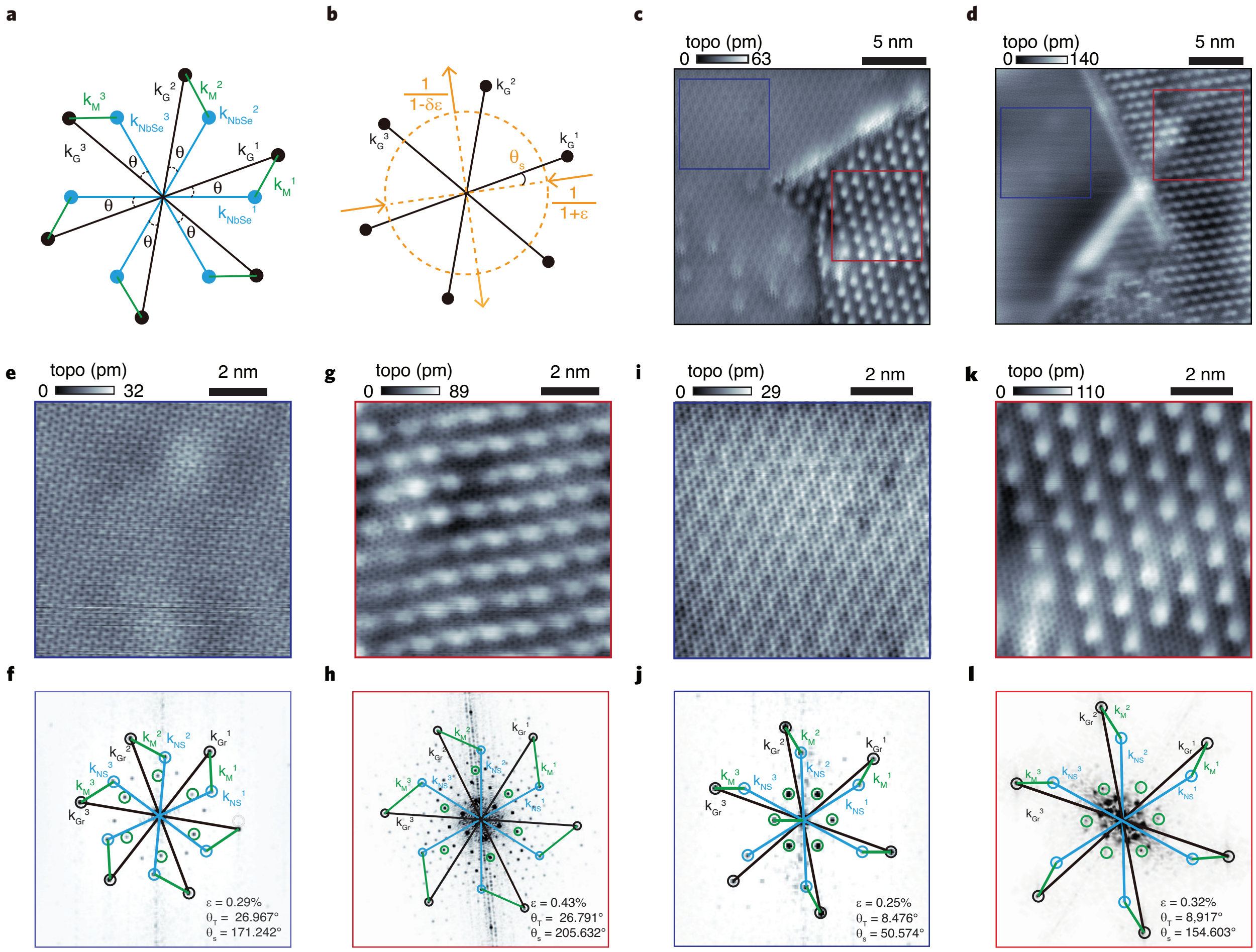

FIG. S3.1. **Experimentally determination of the strain in the Gr/2H-NbSe$_2$ and Gr/1T-NbSe$_2$ heterostructures.** **a,** Reciprocal-space construction of the moiré lattice. The NbSe$_2$ layer is rotated with respect to the graphene sheet by a twist angle of θ_t. The lattice mismatch between NbSe$_2$ and graphene results in a new periodicity, characterised by the reciprocal lattice vectors of the moiré connecting $\boldsymbol{k}_{\mathrm{G}}^{\ell}$ and $\boldsymbol{k}_{\mathrm{NbSe}}^{\ell}$. **b,** Schematic illustration of applying an uniaxial heterostrain to the graphene sheet at an angle θ_s with respect to $\boldsymbol{k}_{\mathrm{G}}^{1}$. **c,d** Constant-current STM images of a phase boundary separating Gr/2H-NbSe$_2$ from Gr/1T-NbSe$_2$ for samples twisted by 9° (c) and 27° (d). **e-h** Atomically resolved subimages of the 9° sample on the Gr/2H-NbSe$_2$ side (e) (indicated by the blue box in panel c) and the Gr/1T-NbSe$_2$ side (g) (indicated by the red box in panel c), together with their corresponding FFTs (f, h). **i-l**, Corresponding subimages (i, k) indicated by the blue and red boxes in panel d and FFTs (j, l) for the 27° sample.

As shown in Fig. S3.1b, applying now an uniaxial heterostrain to the graphene layer along an arbitrary direction rotated by an angle of θ_s from the $\boldsymbol{k}_{\mathrm{G}}^{(1)}$ direction can be represented by a strain

matrix $\mathbf{S}$:

$$\mathbf{S}(\epsilon, \theta_s) = \mathbf{R}(-\theta_s)\mathbf{E}(\epsilon)\mathbf{R}(\theta_s).$$

Here, $\mathbf{R}(\theta_s)$ is the rotation matrix, and $\mathbf{E}(\epsilon)$ is the strain matrix when the strain is along $\boldsymbol{k}_{\mathrm{G}}^{(1)}$ direction (θ_s = 0):

$$\mathbf{S}(\epsilon, 0) = \mathbf{E}(\epsilon) = \begin{bmatrix} 1/(1+\epsilon) & 0 \\ 0 & 1/(1-\delta\epsilon) \end{bmatrix},$$

where ϵ is the strain strength and δ is the Poisson ratio of the material ($\delta = 0.16$ for graphene).

As a result, the moiré wavevector with strain is modified to

$$\boldsymbol{k}_{\mathrm{M,s}}^1 = \mathbf{S}(\epsilon, \theta_s)\boldsymbol{k}_{\mathrm{G}}^1 - \boldsymbol{k}_{\mathrm{NbSe}}^1, \quad \boldsymbol{k}_{\mathrm{M,s}}^2 = \mathbf{S}(\epsilon, \theta_s)\boldsymbol{k}_{\mathrm{G}}^2 - \boldsymbol{k}_{\mathrm{NbSe}}^2, \quad \boldsymbol{k}_{\mathrm{M,s}}^3 = \mathbf{S}(\epsilon, \theta_s)\boldsymbol{k}_{G}^3 - \boldsymbol{k}_{\mathrm{NbSe}}^3.$$

From the FFTs in Fig. S3.1f, h, j and l, we obtain the experimental length of moiré wavevector in three directions $\boldsymbol{k}_{\mathrm{exp}}^{(\ell)}$ ($\ell = 1, 2, 3$). We determine the strain magnitude ε, the interlayer twist θ_{t} and the strain direction θ_{s} by nonlinear least squares minilization:

$$\min_{\varepsilon,\theta,\theta_{\mathrm{s}}} \sum_{\ell=1}^{3} \left[|\boldsymbol{k}_{\mathrm{s}}^{(\ell)}(\varepsilon, \theta, \theta_{\mathrm{s}})| - |\boldsymbol{k}_{\mathrm{exp}}^{(\ell)}| \right]^2, \quad \text{(S3.4)}$$

The extracted heterostrain for the two structural phases in both samples is summarised in Table S3.1. The values we found are comparable to the strain strength $\epsilon = 0.68\%$ in magic angle twist bilayer graphene [4] and slightly larger than $\epsilon = 0.03\%$ in twist double bilayer graphene [3].

TABLE S3.1. Summary of the heterostrain analysis for both samples and different regions

Region	ε (%)	θ_{t} (°)	θ_{s} (°)
2×2 (9°), Gr/2H-$NbSe_2$	0.25	8.476	50.574
2×2 (9°), Gr/1T-$NbSe_2$	0.32	8.917	154.603
$\sqrt{3}\times\sqrt{3}$ R30° (27°), Gr/2H-$NbSe_2$	0.29	26.967	171.242
$\sqrt{3}\times\sqrt{3}$ R30° (27°), Gr/1T-$NbSe_2$	0.43	26.791	205.632

B. Local relaxation across the 2H/1T phase boundary

The phase-boundary scan frames of Fig. S3.1c,d contain graphene/2H-$NbSe_2$ and graphene/1T-$NbSe_2$ regions of the same device in a single image, acquired with the same tip and imaging conditions. To quantify the proximity to commensurability, we evaluate the mismatch function

$f(\theta)$ from Eq. (2) at the measured twist angles. Its minimum $f \approx 0.452|\mathbf{a}_{\rm Gr}|$ for 2×2 at $\theta \approx 9.1^\circ$ and $f \approx 0.155|\mathbf{a}_{\rm Gr}|$ for $\sqrt{3} \times \sqrt{3}$ R30° at $\theta \approx 26.9^\circ$. This value defines the irreducible lattice incommensurability, removable only by the isotropic strains shown in Fig. 1c–f. The excess above this value, therefore, quantifies the mismatch due to angular misalignment.

For the 9° device, the as-stacked 2H region ($\theta = 8.48^\circ$) has $f \approx 0.469|\mathbf{a}_{\rm Gr}|$, i.e. an mismatch of $0.018|\mathbf{a}_{\rm Gr}|$, whereas the converted 1T region ($\theta = 8.92^\circ$) has $f \approx 0.454$, i.e. an excess of only $0.003|\mathbf{a}_{\rm Gr}|$: the 2H→1T conversion reduces the excess mismatch by a factor of about seven, relaxing the local geometry towards the optimal angle. For the 27° device, the as-stacked angle ($\theta = 26.97^\circ$) already coincided with the optimum within 0.07°, and the 1T region ($\theta = 26.79^\circ$) remains at the optimum within the experimental uncertainty; here, no angular relaxation was required. Including the measured uniaxial heterostrain of Table S3.1 changes these f-values by less than 2% of the graphene lattice constants and does not affect the conclusion. The relaxation of the local twist angle towards the commensurate condition upon formation of the metastable 1T phase is a direct manifestation of the energetic lock-in of the registry.

S4. TOLERANCE OF THE FOLDING CONFIGURATIONS TO TWIST-ANGLE DEVIATIONS

The measured twist angles (Table S3.1) differ slightly from the near-commensurate minima at $\theta_0 \approx 9.1°$ (2×2) and $\theta_0 \approx 26.9°$ ($\sqrt{3} \times \sqrt{3}$ R30°). The relevant question is therefore, whether the deviation of $\delta\theta \approx 0.1°$ is small enough to leave the folding assignment and the local registry that underlies it intact.

A. Sensitivity of the mismatch function

We derive the angular sensitivity directly from the definition of the mismatch function (Eq. (2) of the main text, Fig. 1b),

$$f(\theta) = \min_{m,n\in\mathbb{Z}} \left| m\,\mathbf{R}(\theta)\,\boldsymbol{a}_{\mathrm{G1}} + n\,\mathbf{R}(\theta)\,\boldsymbol{a}_{\mathrm{G2}} - j\,\boldsymbol{a}_{\mathrm{CDW1}} - k\,\boldsymbol{a}_{\mathrm{CDW2}} \right| . \tag{S4.1}$$

For convenience, we define the rotated graphene supercell vector and the fixed target CDW vector as

$$\boldsymbol{G}(\theta) = \mathbf{R}(\theta)\,(m\,\boldsymbol{a}_{\mathrm{G1}} + n\,\boldsymbol{a}_{\mathrm{G2}}) \quad \text{and} \quad \boldsymbol{T} = j\boldsymbol{a}_{\mathrm{CDW1}} + k\boldsymbol{a}_{\mathrm{CDW2}}, \tag{S4.2}$$

so that $f(\theta) = \min_{m,n} |\boldsymbol{G}(\theta) - \boldsymbol{T}|$. The law of cosines gives

$$f^2(\theta) = G^2(\theta) + T^2 - 2\,|G(\theta)|\,|T|\cos\phi(\theta)\,, \tag{S4.3}$$

where $\phi(\theta)$ is the angle between $\boldsymbol{G}(\theta)$ and $\boldsymbol{T}$.

For a small deviation $\delta\theta$ around a given minimum, the minimising integers (m, n, j, k) remain the same, so the difference of Eq. (S4.3) reads

$$\begin{aligned} f^2(\theta+\delta\theta) - f^2(\theta) =& \big[G^2(\theta+\delta\theta) - G^2(\theta)\big] + \big[T^2 - T^2\big] \\ &- 2|T|\big[|G(\theta+\delta\theta)|\cos\phi(\theta+\delta\theta) - |G(\theta)|\cos\phi(\theta)\big]\,. \end{aligned} \tag{S4.4}$$

As rotations preserve length, so $|G(\theta)| = |G(\theta+\delta\theta)|$ the first and second terms vanish. Moreover, $\mathbf{R}(\theta)$ rotates $\boldsymbol{G}$ rigidly, so $\phi(\theta+\delta\theta) = \phi(\theta) + \delta\theta$. Equation (S4.4) therefore collapses to

$$f^2(\theta+\delta\theta) - f^2(\theta) = -2|G||T|\big[\cos\!\big(\phi(\theta)+\delta\theta\big) - \cos\phi(\theta)\big]\,. \tag{S4.5}$$

At the optimal angle θ_0, f is minimal, equation S4.3 is minimal, which requires $\phi(\theta_0) = 0$: the rotated graphene vector is then collinear with the target CDW superlattice. Setting $\phi(\theta_0) = 0$ in Eq. (S4.5) gives

$$f^2(\theta_0+\delta\theta) - f_0^2 = 2|G||T|\,(1-\cos\delta\theta) \simeq GT\,\delta\theta^2\,, \tag{S4.6}$$

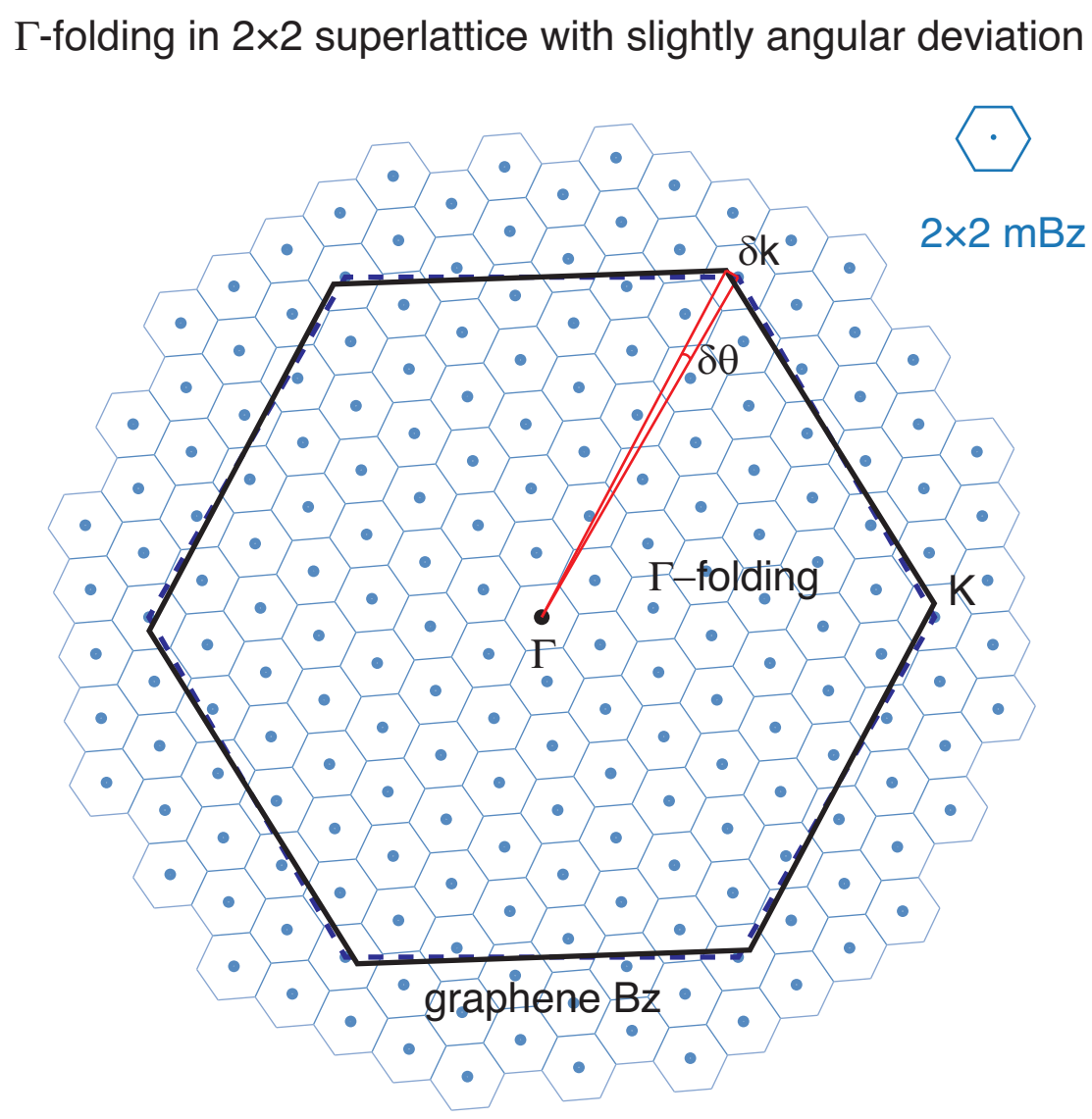

FIG. S4.1. **Reciprocal-space robustness of the Γ-folding to a twist-angle deviation.** The graphene Brillouin zone (black hexagon) is tiled by the 2×2 mini-Brillouin zones (mBZ, blue) into which it folds for the 2×2 configuration. A twist-angle deviation $\delta\theta$ rotates the graphene lattice rigidly and displaces the folded image slightly.

Factoring the left-hand side as a difference of squares, $f^2(\theta_0 + \delta\theta) - f_0^2 = [f(\theta_0 + \delta\theta) - f_0]\,[f(\theta_0 + \delta\theta) + f_0]$, and noting that $f(\theta_0 + \delta\theta) \to f_0$ as $\delta\theta \to 0$ so that $f(\theta_0 + \delta\theta) + f_0 \simeq 2f_0$, we obtain the leading angular deviation of the mismatch function,

$$\Delta f = f(\theta_0 + \delta\theta) - f(\theta_0) = \frac{f^2(\theta_0 + \delta\theta) - f_0^2}{f(\theta_0 + \delta\theta) + f_0} \simeq \frac{|G||T|\,\delta\theta^2}{2f_0}\,, \tag{S4.7}$$

For the 2×2 configuration, we can approximate $|T| \approx |G| = \sqrt{111}|a_{\mathrm{Gr}}|$, $f_0 = 0.15|a_{\mathrm{Gr}}|$, and $\delta\theta = 0.1^\circ$, gives $\Delta f = 0.001|a_{\mathrm{Gr}}|$, which is very small and can be neglected in both experiment and theoretical modelling. Similarly, the $\sqrt{3} \times \sqrt{3}\,\mathrm{R}30^\circ$ configuration, we can approximate $|T| \approx |G| = \sqrt{79}|a_{\mathrm{Gr}}|$, $f_0 = 0.1|a_{\mathrm{Gr}}|$, and $\delta\theta = 0.1^\circ$, gives $\Delta f = 0.001|a_{\mathrm{Gr}}|$.

B. Robustness of the Γ/K folding against the rotational mismatch

We now show that the band-folding assignment of Sec. S8 is robust against the small twist-angle deviation considered here. Following Eq. (S8.1), the commensurate superlattice that links the graphene lattice to the CDW supercell is spanned by the two vectors

$$\boldsymbol{L}_1 = n\,\boldsymbol{a}_1 + m\,\boldsymbol{a}_2\,, \qquad \boldsymbol{L}_2 = \mathbf{R}(60^\circ)\,\boldsymbol{L}_1 = -m\,\boldsymbol{a}_1 + (n + mFactoring)\,\boldsymbol{a}_2\,, \tag{S4.8}$$

where $\boldsymbol{L}_2$ is fixed by the C_3 symmetry of the hexagonal cell. For the 2×2 configuration $(n,m) = (11,-1)$, so the first superlattice vector is $\boldsymbol{L}_1 = 11\,\boldsymbol{a}_1 - \boldsymbol{a}_2$ and the second follows from Eq. (S4.8) as $\boldsymbol{L}_2 = \boldsymbol{a}_1 + 10\,\boldsymbol{a}_2$. For the $\sqrt{3}\times\sqrt{3}$ R30° configuration $(n,m) = (3,7)$, we find $\boldsymbol{L}_1 = 3\,\boldsymbol{a}_1 + 7\,\boldsymbol{a}_2$ and $\boldsymbol{L}_2 = -7\,\boldsymbol{a}_1 + 10\,\boldsymbol{a}_2$.

A twist-angle deviation $\delta\theta$ rotates the graphene lattice rigidly through the rotation matrix

$$\mathbf{R}(\delta\theta) = \begin{pmatrix} \cos\delta\theta & -\sin\delta\theta \\ \sin\delta\theta & \cos\delta\theta \end{pmatrix}. \tag{S4.9}$$

The real-space graphene lattice vectors transform by the angular misalignment as

$$A_\mathrm{G} \to A'_\mathrm{G} = \mathbf{R}(\delta\theta)\,A_\mathrm{G}, \qquad \text{equivalently} \qquad {A'_\mathrm{G}}^{\mathsf{T}} = A_\mathrm{G}^{\mathsf{T}}\,\mathbf{R}(\delta\theta)^{\mathsf{T}}. \tag{S4.10}$$

so the graphene reciprocal vectors transform as

$$B_\mathrm{G} \to B'_\mathrm{G} = \mathbf{R}(\delta\theta)^{-\mathsf{T}} B_\mathrm{G}, \qquad \mathbf{R}(\delta\theta)^{-\mathsf{T}} = \mathbf{R}(\delta\theta). \tag{S4.11}$$

Due to the angular misalignment, the K points of the graphene move by

$$\delta\boldsymbol{k} = \mathrm{K}' - \mathrm{K} = (\mathbf{R}(\delta\theta) - I)\,\mathrm{K}, \tag{S4.12}$$

Expanding to first order in the small twist mismatch $\delta\theta \ll 1$, $\mathbf{R}(\delta\theta) \approx I + \delta\theta\,\mathbf{J}$ with $\mathbf{J} = \left(\begin{smallmatrix} 0 & -1 \\ 1 & 0 \end{smallmatrix}\right)$, so that

$$\delta\boldsymbol{k} \approx \delta\theta\,\mathbf{J}\mathrm{K}. \tag{S4.13}$$

The displacement is purely tangential ($\perp$ K, since $\mathbf{J}$ rotates K by 90°), with magnitude $|\delta\boldsymbol{k}| = 2|\mathrm{K}|\sin(\Delta\theta/2) \approx |\mathrm{K}|\,\delta\theta$. Expressed as a fraction of the mBZ reciprocal vector $|\boldsymbol{b}_\mathrm{S}| = 4\pi/(\sqrt{3}\,|\boldsymbol{a}_\mathrm{SL}|)$, and using $|\mathrm{K}| = 4\pi/(3a_\mathrm{G})$, For the 2×2 superlattice

$$\frac{|\delta\boldsymbol{k}|}{|\boldsymbol{b}_\mathrm{S}|} = \frac{|\boldsymbol{a}_\mathrm{SL}|}{\sqrt{3}\,a_\mathrm{G}}\,\delta\theta \approx 5.8\,\delta\theta \qquad \big(2\times2,\ |\boldsymbol{a}_\mathrm{SL}| = 2|\boldsymbol{a}_\mathrm{CDW}| \approx 2.48\,\mathrm{nm}\big). \tag{S4.14}$$

For the experimental twist deviation $\delta\theta \approx 0.1° = 1.7\times10^{-3}\,\mathrm{rad}$, this gives $|\delta\boldsymbol{k}|/|\boldsymbol{b}_\mathrm{S}| \approx 1.0\%$: the Dirac point is displaced from Γ by only about 1% of a mBZ reciprocal vector. The K-folding ($\sqrt{3}\times\sqrt{3}$ R30°) case follows identically, with the slightly smaller prefactor $|\boldsymbol{a}_\mathrm{SL}|/(\sqrt{3}\,a_\mathrm{G}) \approx 5.0$ ($|\boldsymbol{a}_\mathrm{SL}| = \sqrt{3}\,|\boldsymbol{a}_\mathrm{CDW}| \approx 2.15\,\mathrm{nm}$), so the $\mathrm{K_m}$ corner image is displaced by $\approx 0.9\%$ at $0.1°$. In conclusion, this small angular deviation of the experimentally achieved sample can be neglected in DFT calculation.

C. Impact on the local stacking registry

By definition, the mismatch function f is the lateral offset by which the graphene–$\sqrt{13} \times \sqrt{13}\,\mathrm{R}13.9°$ registry fails to repeat after one supercell vector $\boldsymbol{a}_{\mathrm{SL}}$: at commensurability $f = 0$, the registry pattern is strictly periodic and a single supercell already includes the complete set of local registries. A twist deviation $\delta\theta$ rotates the graphene lattice rigidly relative to the fixed CDW, so the registry drifts by

$$\delta r \simeq |\boldsymbol{a}_{\mathrm{SL}}|\,\delta\theta \tag{S4.15}$$

across one supercell. For $\delta\theta = 0.1°$ this is $\delta r \approx 0.018\,a_{\mathrm{G}}$ (2×2) and $0.015\,a_{\mathrm{G}}$ ($\sqrt{3}\times\sqrt{3}\,\mathrm{R}30°$), i.e. below $5\,\mathrm{pm}$; the offset of any atom from its commensurate registry with a single cell is smaller still. This is negligible in both experimental STM measurement and the DFT cacultion, as this value is much smaller compare with the C–C distance (1.42 Å), and the CDW period ($|\boldsymbol{a}_{\mathrm{CDW}}| = 12.4$ Å), so within each supercell the local environments are indistinguishable from their commensurate counterparts. The residual incommensuration manifests only as an extremely slow lock registry mismatch accumulating over tens of supercells.

S5. WIDE RANGE STS SPECTROSCOPY

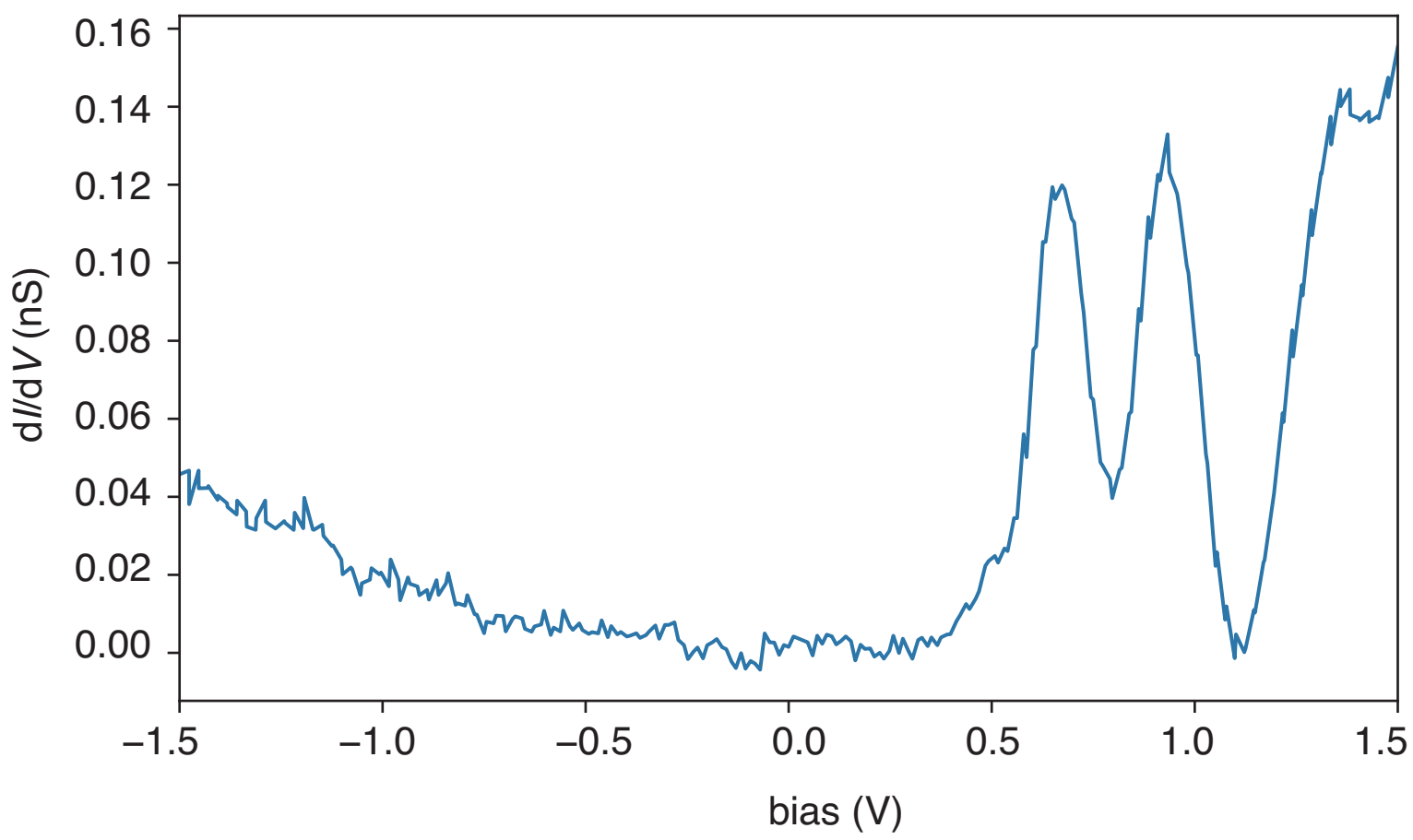

FIG. S5.1. **Wide range dI/dV spectroscopy taken at the A-site of Gr/1T-NbSe$_2$** ($V = 1.5$ V, $I = 100$ pA, $V_{\mathrm{mod}} = 10$ mV). Pronounced conductance resonances are located on the positive-bias side, while the negative-bias side remains featureless. For this reason, the main manuscript concentrates on the positive-bias regime, where all electronically relevant features are located.

S6. DETERMINATION OF THE PEAK POSITIONS

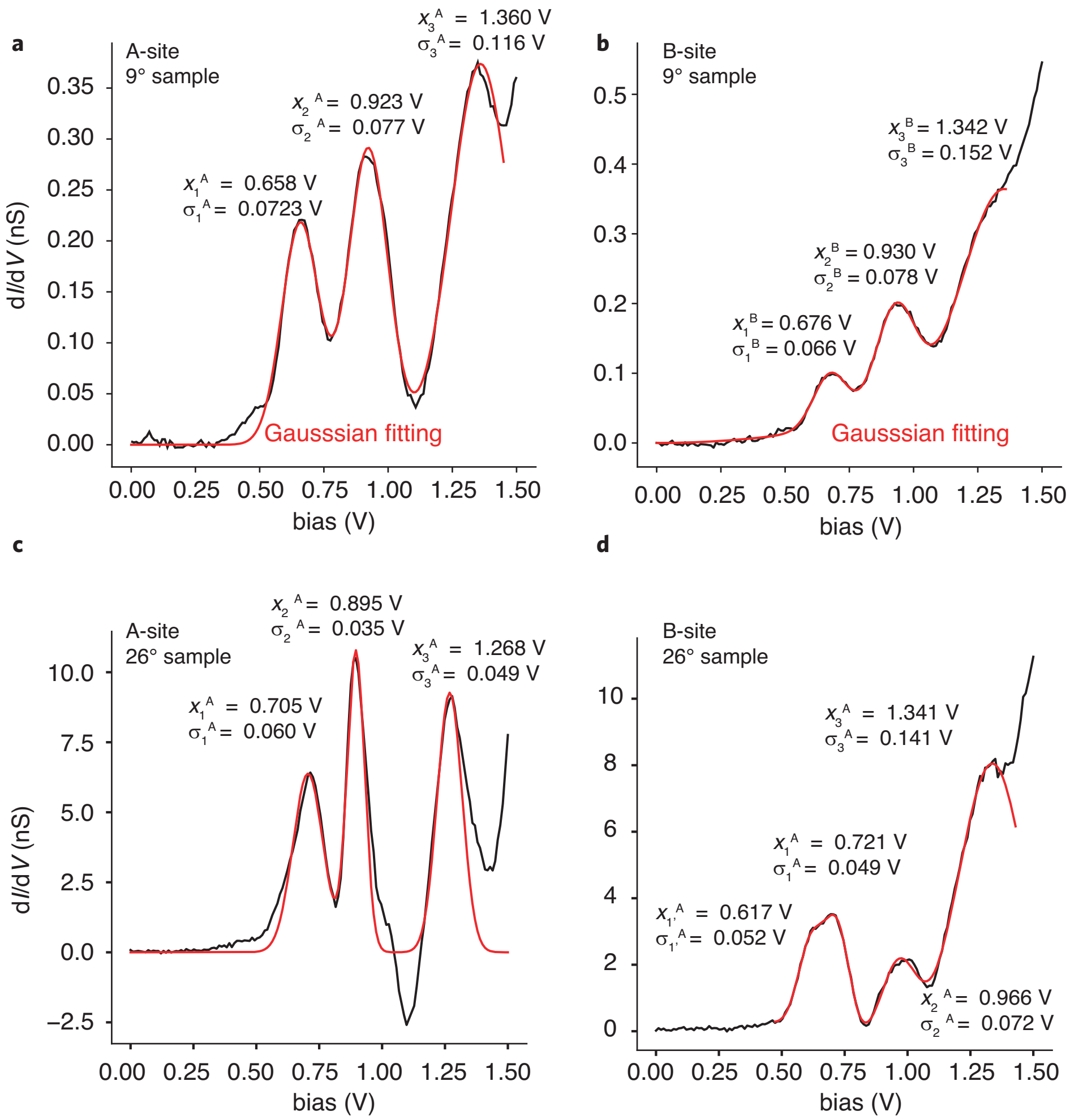

FIG. S6.1. **Examples of the fittings (red) of the dI/dV spectra (black) at the A-site and B-site.** To quantify the characteristic energy scales, we used the sum of 3 Gaussians and a quadratic background to fit the differential conductance spectra. The $x_{\mathrm{pk},i}$ mark the peak centres with the corresponding energies $E_{\mathrm{pk},i} = e\, x_{\mathrm{pk},i}$, and σ_i the corresponding Gaussian widths.

.

S7. EXTRACTION OF THE FORM FACTOR

To quantify the observed rotational symmetry breaking in our dI/dV grid measurements, we determine the complex CDW form factors $\psi_i = A_i e^{i\theta_i}$ at each bias, where the amplitudes A_i and phases θ_i are associated with the CDW wavevectors $\boldsymbol{Q}_{1,2,3}$.

This supplementary note details the extraction procedure. To illustrate the fundamental principles involved, we begin by considering a one-dimensional CDW, and then extend this to the triangular triple-$\boldsymbol{q}$ CDW relevant to 1T-$NbSe_2$.

A. 1D illustration

A 1D CDW that modulates the LDOS, $\rho(r)$, can be expressed as a plane wave with a finite background [5]:

$$\rho\left(r\right) = A\cos\left(Q\cdot r+\theta\right)+\rho_0 = \frac{1}{2}\left(Ae^{i(Q\cdot r+\theta)}+A_i e^{-i(Q\cdot r+\theta)}\right)+A_0,$$

where A is the amplitude of the modulation, θ its phase, and Q is the reciprocal vector of the CDW. Thus, the modulation of the CDW can be further expressed as the complex form factor $\psi = A\exp(i\theta)$ multiplied by a plane wave function $\exp\left(iQ\cdot r\right)$ [6]:

$$\rho\left(r\right) = \frac{1}{2}\left(\psi e^{iQ\cdot r}+\psi^* e^{-iQ\cdot r}\right)+A_0.$$

By applying the Fourier transformation to $\rho(r)$, we obtain

$$\begin{aligned}\tilde{\rho}\left(k\right) &= \int dr\ \exp\left(-ik\cdot r\right)\rho\left(r\right)\\ &= \frac{1}{2}\left[\int dr\,\psi\exp\left(i\left(Q-k\right)\cdot r\right)+\int dr\,\psi^*\exp\left(i\left(-Q-k\right)\cdot r\right)\right]\\ &= \frac{(2\pi)^1}{2}\left[\psi\delta(Q-k)+\psi^*\delta(-Q-k)\right].\end{aligned}$$

Thus, the Bragg peak at $k = Q$ in the reciprocal space hold the information of complex factor ψ and the other Bragg peak at $k = -Q$ holds its complex conjugate ψ^*.

B. Extension to 2D triple-q CDW

In our 2D system, a triple $\boldsymbol{q}$-CDW forms a triangular superlattice, and the charge modulates along the three equivalent directions defined by wavevectors $\boldsymbol{Q}_1$, $\boldsymbol{Q}_2$, and $\boldsymbol{Q}_3$ [7]. These three

wavevectors are equivalent in magnitude and rotated by $120°$. Consequently, the resulting LDOS modulation is the incoherent sum of three plane waves [8]:

$$\rho(\boldsymbol{r}) = \sum_{i=1}^{3} \rho_i + \rho_0 = \frac{1}{2} \sum_{i=1}^{3} \left(\psi_i e^{i\boldsymbol{Q}_i \cdot \boldsymbol{r}} + \psi_i^* e^{-i\boldsymbol{Q}_i \cdot \boldsymbol{r}} \right) + A_0. \tag{S7.1}$$

Equation S7.1 indicates that for fixed $\boldsymbol{Q}_i$, all spatial various are registered by the three form factors (ψ_1, ψ_2, ψ_3). Two methods can be used to extract the form factors from the STS grids. The first method is to fit the STS grid data by minimizing the difference between the observed STS grids and the equation S7.1 [8, 9], i. e.:

$$\min_{\{\psi_1, \psi_2, \psi_3\}} \left(\text{STS} - \rho(\psi_1, \psi_2, \psi_3)\right)^2 .$$

The second method is to apply the Fourier transformation to equation S7.1:

$$\begin{aligned}
\tilde{\rho}(\boldsymbol{k}) &= \int d\boldsymbol{r}\, \exp(-i\boldsymbol{k} \cdot \boldsymbol{r})\, \rho(\boldsymbol{r}) \\
&= \frac{1}{2} \sum_{i=1}^{3} \left[\int d\boldsymbol{r}\, \psi_i \exp\left(i\left(\boldsymbol{Q}_i - \boldsymbol{k}\right) \cdot \boldsymbol{r}\right) + \int d\boldsymbol{r}\, \psi_i^* \exp\left(i\left(-\boldsymbol{Q}_i - \boldsymbol{k}\right) \cdot \boldsymbol{r}\right) \right] \\
&= \frac{(2\pi)^2}{2} \sum_{i=1}^{3} \left[\psi_i \delta(\boldsymbol{Q}_i - \boldsymbol{k}) + \psi_i^* \delta(-\boldsymbol{Q}_i - \boldsymbol{k}) \right].
\end{aligned} \tag{S7.2}$$

This Fourier transformation shows that the form factors ψ_i appear at $\boldsymbol{k} = \boldsymbol{Q}_i$ corresponding to the Bragg peaks, and their complex conjugates ψ^* appear at centrosymmetric counterparts $\boldsymbol{k} = -\boldsymbol{Q}_i$. In practice, the extraction of the form factors from the FFT of the STS grid needs a Gaussian window [10]:

$$\psi_i = \int_{\boldsymbol{k} \in P(\boldsymbol{Q}_i)} d\boldsymbol{k} \int d\boldsymbol{r}\, \exp(-i\boldsymbol{k} \cdot \boldsymbol{r})\, \text{STS}(\boldsymbol{r})\, w(\boldsymbol{r}), \tag{S7.3}$$

where $\omega(\boldsymbol{r}) = \exp\left(-\boldsymbol{r}^2/2R^2\right)$ is a Gaussian window function and $P(\boldsymbol{Q}_i)$ represent appropriately sized square area around Bragg spot $\boldsymbol{Q}_i$. The form factors are complex numbers; thus, to visualise them, we decomposed them into their magnitude and phase and show them in separate images. In the FFT phase images, the colour of each pixel represents the phase of the FFT patterns, while their brightness indicates their magnitude. For clarity, pixels with magnitudes smaller than 10% are filtered out to be white. The uniform colour around the Bragg peaks indicates that the phase takes on a constant value within each Bragg peak.

The FFT magnitude and phase patterns reveal six Bragg spots forming a hexagonal pattern. According to the equation S7.2, we focus on the three Bragg peaks that are separated by $120°$ rotations, as they correspond to the form factors ψ. The remaining three centrosymmetric counterparts encode the complex conjugates ψ^* as shown in Figs. S7.1 and S7.2.

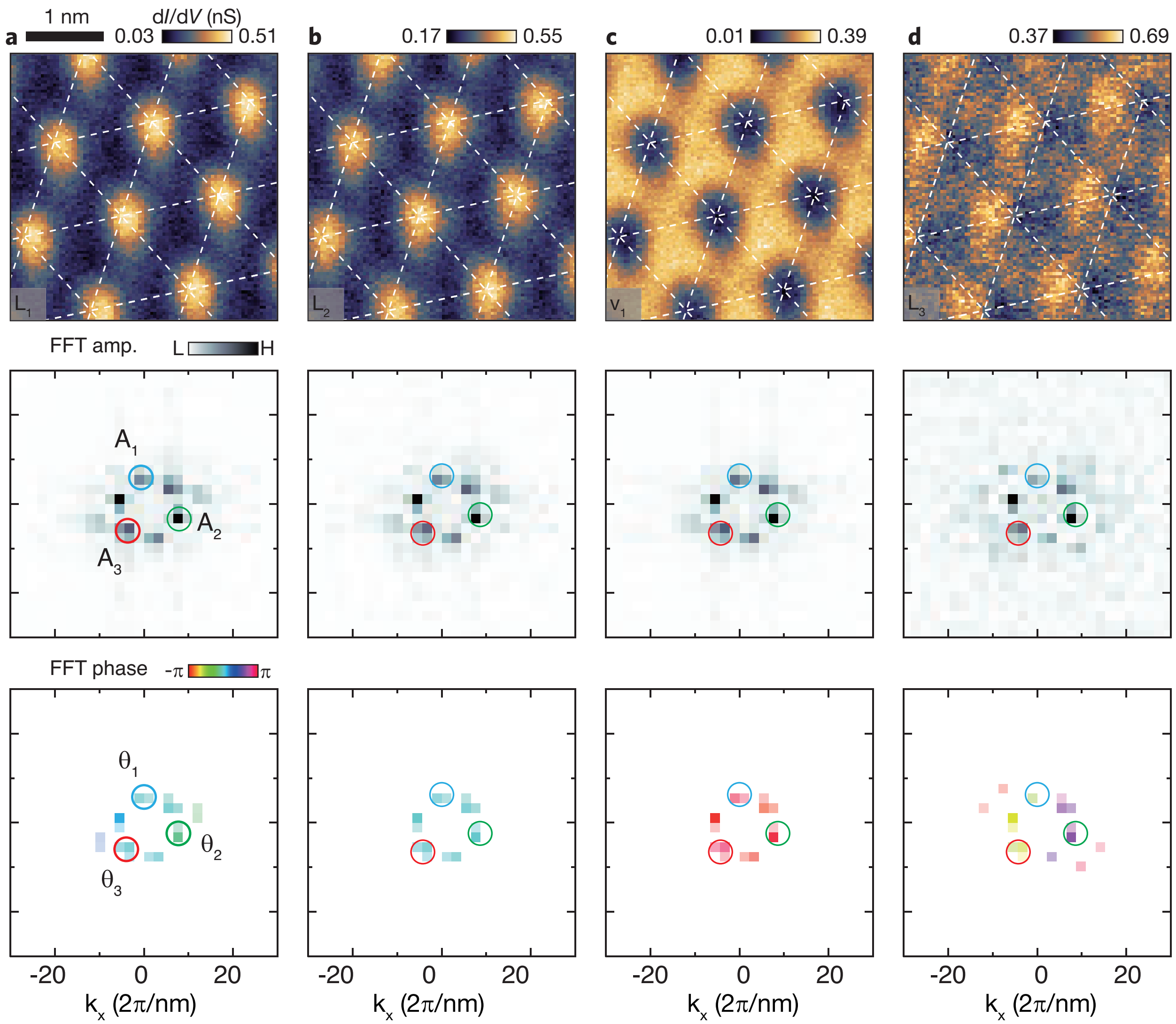

FIG. S7.1. **STS grid measurement of the 9° sample with the corresponding FFT amplitudes and FFT phases.** **a,** slice at the energy of $L_1 = 0.66$ eV, **b,** at $L_2 = 0.92$ eV, **c**, at $v_1 = 1.14$ eV , and **d** at $L_3 = 1.36\ eV$.

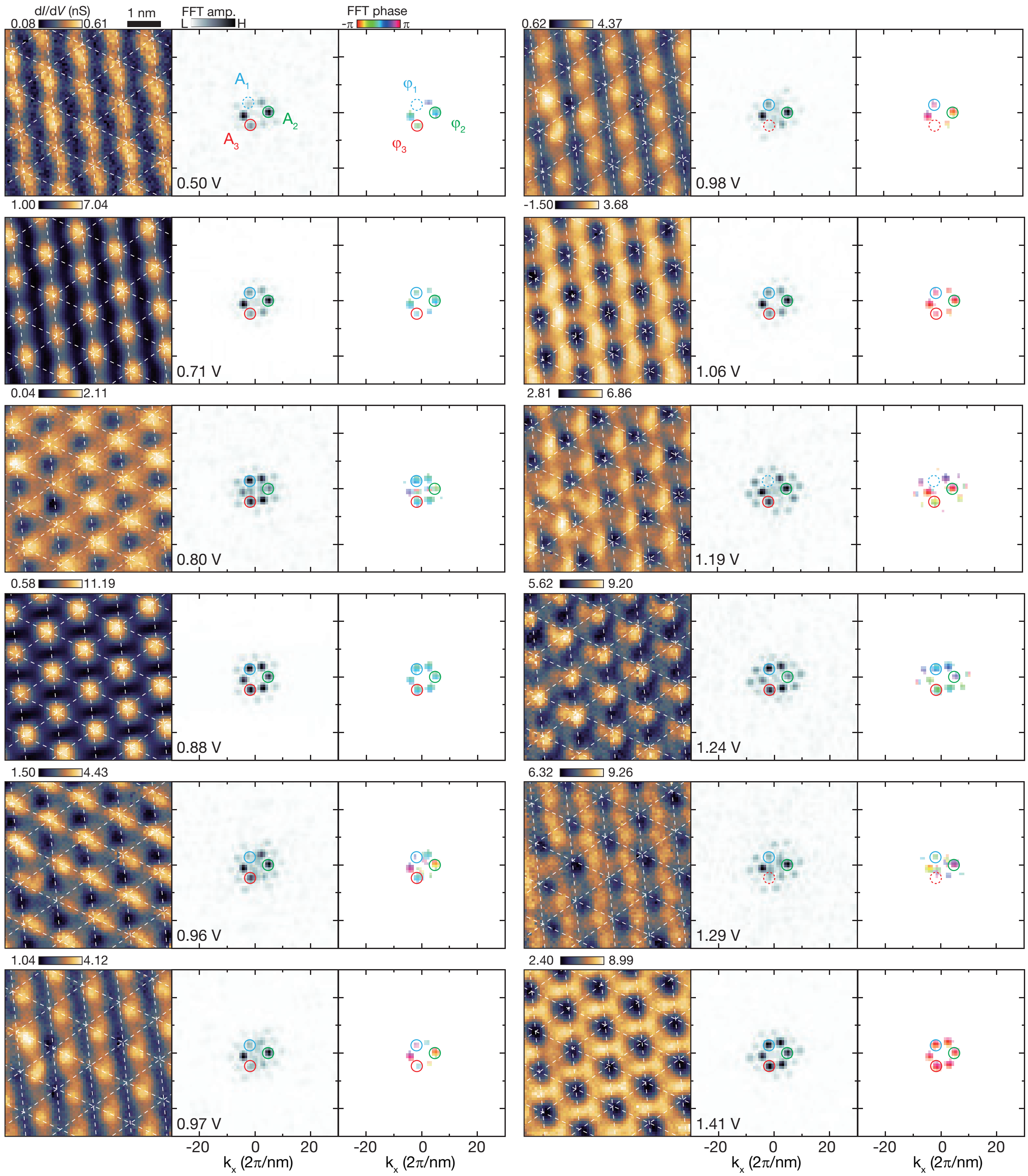

FIG. S7.2. **STS grid measurement of the 27° sample with the corresponding FFT amplitudes and FFT phases**

C. Extraction of the form factor from the calculated LDOS maps

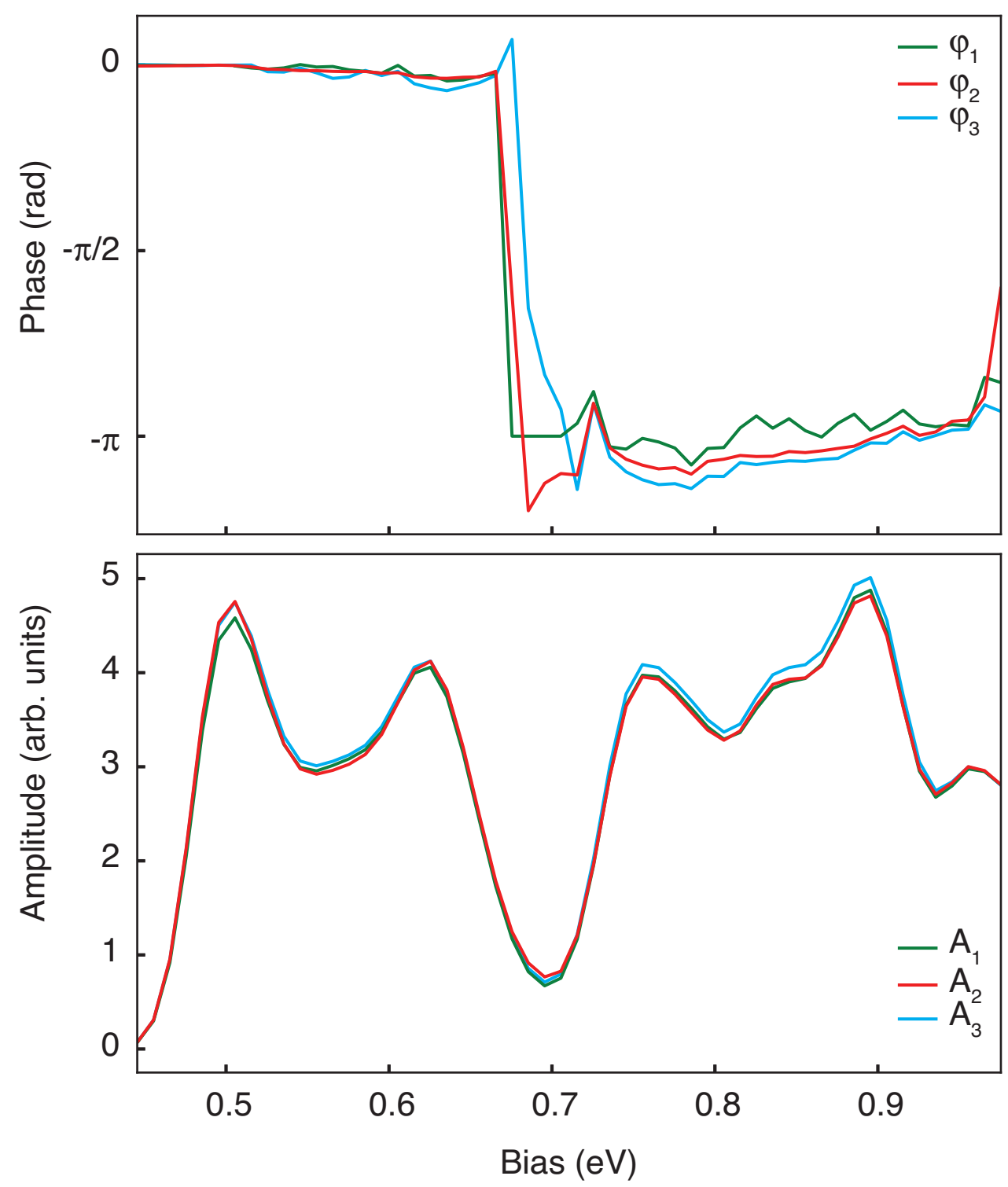

FIG. S7.3. **FFT phases and amplitudes extracted from the calculated LDOS maps of the** $\sqrt{3}\times\sqrt{3}\,\mathrm{R}30^{\circ}$ **superlattice.** The phase analysis is the same as shown in the main text Fig. 3o.

S8. BAND-FOLDING ANALYSIS OF THE 2×2 AND $\sqrt{3}\times\sqrt{3}$ R30° SUPERLATTICES

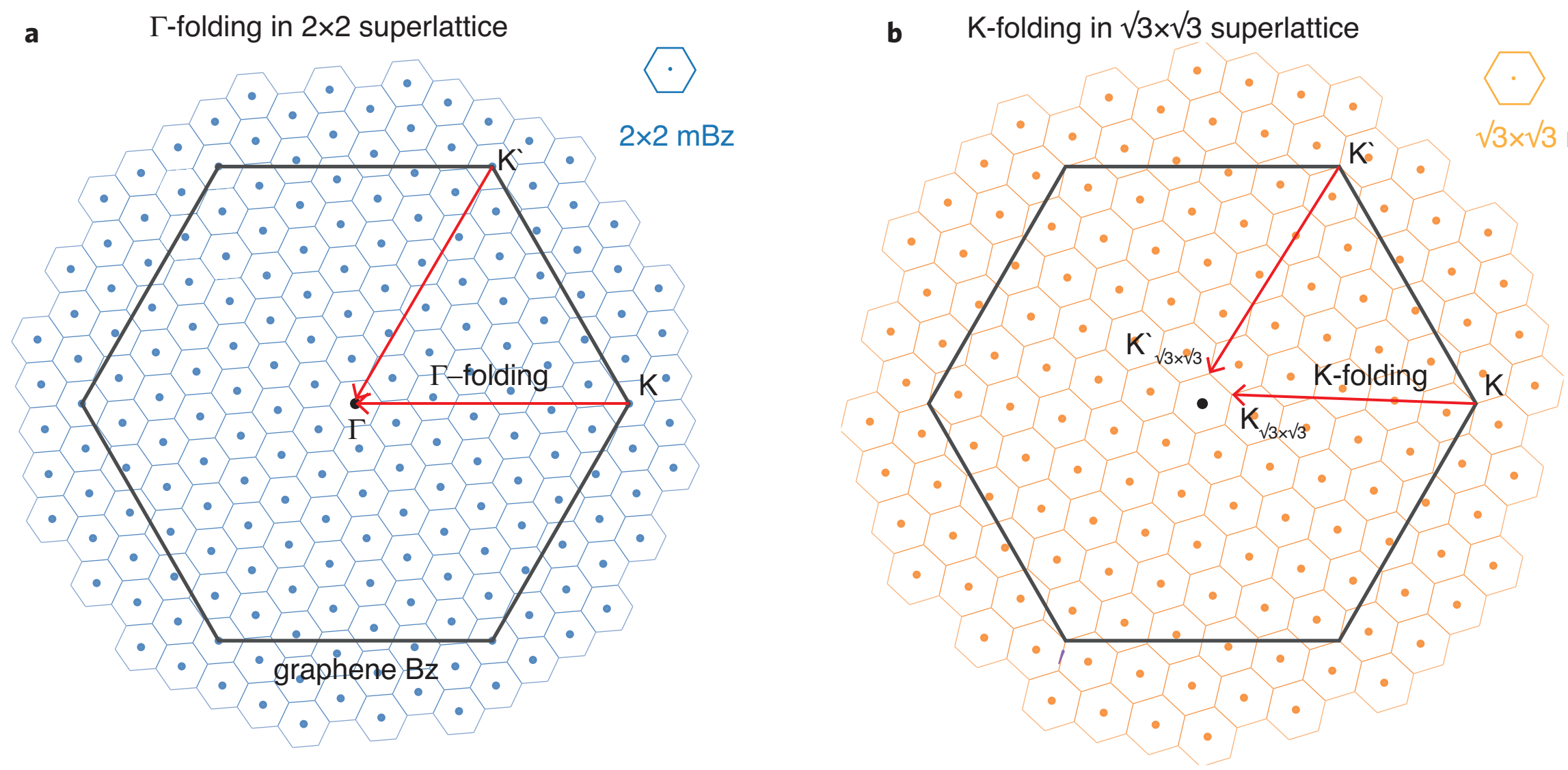

FIG. S8.1. **Reciprocal-space construction of the two band-folding configurations. a,** graphene Brillouin zone (black hexagon) overlaid with the mini-Brillouin-zone (mBZ) tiling of the 2×2 superlattice (blue). The graphene Dirac point at the zone corner coincides with a mBZ centre Γ_{m} after translation by a superlattice reciprocal vector (red arrow), realising Γ-folding. **b,** same construction for the $\sqrt{3}\times\sqrt{3}$ R30° superlattice (orange). Here, the graphene Dirac point coincides with a mBZ corner, so the K and K′ valleys fold to the inequivalent corners $\mathrm{K_m}$ and $\mathrm{K'_m}$ (K-folding), preserving the valley distinction.

Here we derive the band-folding assignment used in the main text directly from the commensuration geometry. The goal is to determine whether the graphene Dirac points are equivalent, in the reduced mini-Brillouin zone (mBZ), to Γ_{m} or to the mBZ corners.

Let $\boldsymbol{a}_1$ and $\boldsymbol{a}_2$ be the primitive graphene lattice vectors, enclosing an angle of 60°, and let $\boldsymbol{b}_1$ and $\boldsymbol{b}_2$ be the reciprocal vectors, with $\boldsymbol{a}_i\cdot\boldsymbol{b}_j = 2\pi\delta_{ij}$. In this convention, one graphene Dirac point is located at $\mathrm{K} = (2\boldsymbol{b}_1+\boldsymbol{b}_2)/3$. A commensurate hexagonal superlattice is spanned by

$$\boldsymbol{L}_1 = n\,\boldsymbol{a}_1 + m\,\boldsymbol{a}_2\,, \qquad \boldsymbol{L}_2 = \mathbf{R}(60^\circ)\,\boldsymbol{L}_1 = -m\,\boldsymbol{a}_1 + (n+m)\,\boldsymbol{a}_2\,, \tag{S8.1}$$

with integer indices (n,m). For the commensurate 2×2 and $\sqrt{3}\times\sqrt{3}$ R30° supercells used in the DFT calculations (Sec. S14), these indices are $(n,m) = (11,-1)$ and $(n,m) = (3,7)$, respectively.

In the reduced-zone scheme, the Dirac point folds to Γ_{m} if and only if its momentum differs from the mBZ centre by a reciprocal lattice vector of the superlattice, $\boldsymbol{G}_{\mathrm{m}}$. Equivalently, K itself

must be a superlattice reciprocal vector, which requires $\mathrm{K} \cdot \boldsymbol{L}_{1,2} \in 2\pi\mathbb{Z}$. Evaluating the scalar products gives

$$\mathrm{K} \cdot \boldsymbol{L}_1 = \frac{2\pi}{3}\,(2n+m)\,, \qquad \mathrm{K} \cdot \boldsymbol{L}_2 = \frac{2\pi}{3}\,(n-m)\,. \tag{S8.2}$$

Because $2n + m \equiv -(n-m) \pmod 3$, the two conditions reduce to the single folding criterion

$$n - m \equiv 0 \pmod 3 \quad \Longleftrightarrow \quad \Gamma\text{-folding.} \tag{S8.3}$$

If $n - m \not\equiv 0 \pmod 3$, K is not a superlattice reciprocal vector and therefore cannot fold to Γ_{m}. Since the superlattice preserves threefold rotational symmetry, the image of K must still be a C_3-invariant momentum of the mBZ. The remaining possibilities are the two inequivalent mBZ corners, $\mathrm{K_m}$ and $\mathrm{K'_m}$ and time-reversal symmetry maps $\mathrm{K}' = -\mathrm{K}$ to the opposite corner.

For the 2×2 superlattice, $(n, m) = (11, -1)$ gives $n - m = 12 \equiv 0 \pmod 3$, so the K and K' valleys are equivalent to Γ_{m} modulo superlattice reciprocal vectors and realise Γ-folding. For the $\sqrt{3} \times \sqrt{3}\ \mathrm{R}30^\circ$ superlattice, $(n, m) = (3, 7)$ gives $n - m = -4 \equiv 2 \pmod 3$, so the K and K' valleys fold to the inequivalent mBZ corners $\mathrm{K_m}$ and $\mathrm{K'_m}$, preserving the valley distinction. Figure S8.1 visualises this construction by tiling reciprocal space with the mBZ of each superlattice.

S9. EXCLUSION OF A TIP-INDUCED ORIGIN OF THE SYMMETRY BREAKING

A directional asymmetry of the probe tip can, in principle, imprint an apparent anisotropy on STM/STS data and must therefore be excluded as a trivial origin of the observed C_3 symmetry breaking. We note that a weak directional tip sensitivity is indeed present in our 2×2 data: it gives rise to small inequalities among the three form-factor amplitudes $A_{1,2,3}$, while the corresponding phases remain degenerate, $\theta_1 = \theta_2 = \theta_3$, over the entire bias range. Importantly, this effect does not alter the underlying symmetry classification, and the C_3 symmetry is therefore preserved in 2×2 (Sec. S7, Fig. S7.1). Below, we provide three independent arguments showing that the symmetry breaking observed in the $\sqrt{3}\times\sqrt{3}\,\mathrm{R}30^\circ$ sample cannot be attributed to such a tip-induced effect.

A. The symmetry-breaking observables are not due to tip directionality

A static directional tip acts, to leading order, as an anisotropic point-spread function, or equivalently as a convolution kernel, in real space. Such a tip anisotropy can rescale the apparent magnitude of a modulation along a preferred direction and may therefore lead to unequal extracted form-factor amplitudes A_i. Crucially, however, it cannot change the relative phases of the CDW components, nor can it create genuinely different local tunnelling spectra at crystallographically equivalent sites.

This distinction is central to our analysis. The evidence for C_3 symmetry breaking in the $\sqrt{3} \times \sqrt{3}\,\mathrm{R}30^\circ$ sample does not rely on amplitude anisotropy, but instead on observables that are insensitive to such a trivial tip-transfer effect: (i) inequivalent form-factor phases, $\theta_1 \neq \theta_2 \neq \theta_3$; (ii) staggered phase jumps occurring at different bias voltages for the three directions; (iii) an additional spectroscopic peak, L_1', appearing at only one of the three B-sites; and (iv) inequivalent dI/dV spectra measured at the three nominally C_3-related B-sites (main text Fig. 3; Sec. S7, Fig. S7.2). These observations cannot be generated by an anisotropic tip transfer function, which can affect signal amplitudes but cannot alter relative phases or introduce site-selective spectroscopic features. The amplitude inequalities A_i, which are the only quantities that a directional tip could plausibly mimic, are therefore explicitly not used as evidence for the symmetry breaking.

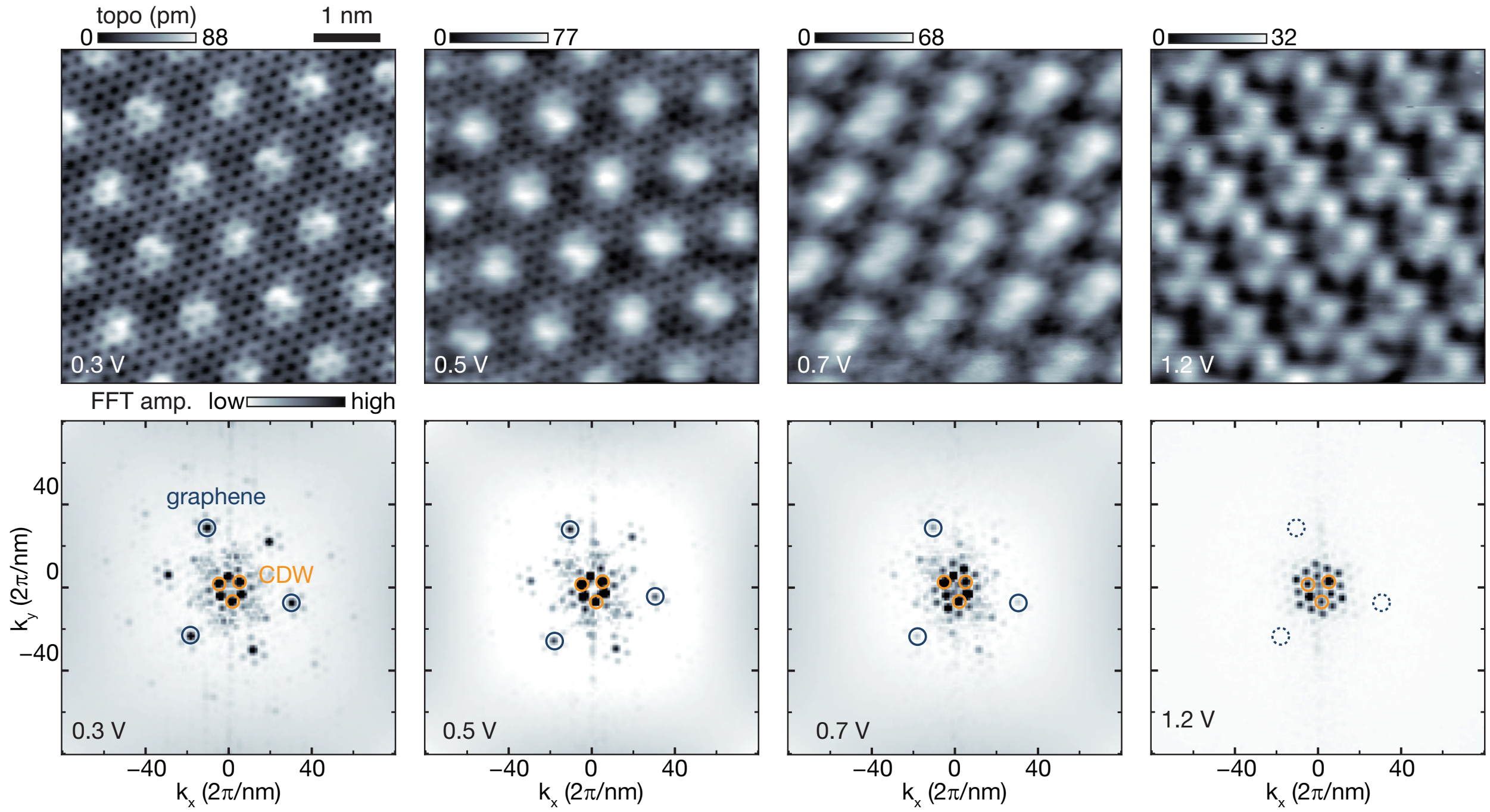

FIG. S9.1. **Bias-dependent constant-current topography of the $\sqrt{3}\times\sqrt{3}$ R30° sample, recorded with a single tip.** STM topographies of the same area at increasing sample bias, each with its corresponding FFT.

B. Bias-dependent topography

Figure S9.1 shows constant-current topographies of the $\sqrt{3} \times \sqrt{3}$ R30° sample recorded at several bias voltages using the *same* tip. At low bias, both the graphene atomic lattice and the CDW superlattice are C_3 symmetric. Upon increasing the bias voltage, the graphene atomic lattice remains undistorted. By contrast, the CDW superlattice becomes increasingly anisotropic and eventually evolves into a pronounced unidirectional stripe pattern near $V = 1.2\,\mathrm{V}$, where the graphene atomic resolution is lost.

This provides a direct internal control against a directional tip artefact. Since the graphene lattice and the CDW-derived superlattice are imaged simultaneously with the same tip, an anisotropic tip transfer function would necessarily act on both periodicities. The selective loss of C_3 symmetry in the CDW-derived component, together with the preserved isotropy of the co-imaged graphene lattice, therefore demonstrates that the observed anisotropy is intrinsic to the CDW-derived electronic structure rather than imposed by the tip.

C. Single-frame comparison across a phase boundary

Finally, the phase-boundary topographies in Fig. S3.1c,d and Fig. S13.1 provide a further internal control. These images contain Gr/2H-$NbSe_2$ and Gr/1T-$NbSe_2$ regions within a single scan frame, and were therefore acquired with the same tip, scanner drift, and imaging conditions. In the Gr/2H-$NbSe_2$ region, both the graphene and $NbSe_2$ atomic lattices are C_3 symmetric. By contrast, in the adjacent Gr/1T-$NbSe_2$ region, the CDW superlattice is distorted, whereas the graphene lattice remains C_3 symmetric. This spatially selective distortion cannot be attributed to tip anisotropy or drift, which would affect both regions within the same scan frame. Instead, it must reflect a property specific to the graphene/1T-$NbSe_2$ system. The C_3-symmetric 2×2 sample, measured with the same instrument, provides an additional in-situ control showing that our tips can faithfully reproduce a threefold-symmetric superlattice.

S10. GEOMETRIC MODEL OF INTERLAYER HYBRIDISATION ANISOTROPY

In the 1T-$NbSe_2$ star-of-David reconstruction, the in-plane clustering of Nb atoms is accompanied by an out-of-plane buckling of the Se sublattice: Se atoms atop the cluster centres are displaced upward relative to those at the cluster boundaries [11]. This vertical modulation directly affects the C-Se interlayer distance and, consequently, the orbital overlap. To capture this effect in our model, we impose a sinusoidal out-of-plane displacement on the top Se atoms. We give a rough estimate on symmetry breaking strength for twisted graphene-$NbSe_2$ (both angles, both in commensurate approximation and for the actual, incommensurate system) in Supplementary Fig. S10.2. To quantify the symmetry breaking, we set up the lattice geometry accordingly and obtain the nearest Se-neighbour for each C-atom in the top graphene layer. The average distance is defined as

$$\boldsymbol{d}_{pp} = \sum_{i,j} \frac{\boldsymbol{r}_{\mathrm{C},i} - \boldsymbol{r}_{\mathrm{Se},j}}{|\boldsymbol{r}_{\mathrm{C},i} - \boldsymbol{r}_{\mathrm{Se},j}|} w(|\boldsymbol{r}_{\mathrm{C},i} - \boldsymbol{r}_{\mathrm{Se},j}|)\,, \tag{S10.1}$$

where $\boldsymbol{r}_{\mathrm{C},i}$ denotes the position of carbon atom i, and $\boldsymbol{r}_{\mathrm{Se},i}$ the position of selenium atom j; the sum runs over all carbon atoms and the top layer of selenium atoms. Motivated by Slater-Koster tight-binding parameters, the weighting function $w(r)$ follows an exponential, i. e., $w(r) = e^{-\alpha r}$. The heuristic parameter α is set to $\alpha = 0.3\,\text{Å}^{-1}$. To mimic the CDW, we displace the Se atoms in the z direction by the following function

$$z(\boldsymbol{r}_{\mathrm{2D}}) = 0.3\,\text{Å} \times \frac{2}{9}\left[\frac{3}{2} + \sum_{i\in\{1,2,3\}} \cos(\boldsymbol{r}_{\mathrm{2D}} \cdot \boldsymbol{G}_i)\right]. \tag{S10.2}$$

A. Connection to microscopic interlayer tunnelling

We start from a two-centre Slater–Koster description of the interlayer Hamiltonian [12], as commonly used for graphene-based moiré systems [13, 14],

$$H_{\mathrm{int}} = \sum_{i,j} t_{zz}(\boldsymbol{r}_{ij}) c_i^\dagger d_j + \mathrm{h.c.}\,, \tag{S10.3}$$

where c_i (d_j) annihilates an electron in a C (Se) p_z orbital and $\boldsymbol{r}_{ij} = \boldsymbol{r}_{\mathrm{C},i} - \boldsymbol{r}_{\mathrm{Se},j}$. The corresponding p_z–p_z hopping is

$$t_{zz}(\boldsymbol{r}) = V_{pp\sigma}(r)\left(\frac{z}{r}\right)^2 + V_{pp\pi}(r)\left[1 - \left(\frac{z}{r}\right)^2\right]\,, \tag{S10.4}$$

with $r = |\boldsymbol{r}|$ and z its out-of-plane component. The radial integrals are short ranged and are commonly represented by exponential functions. Equation (S10.1) is obtained as a coarse-grained

first angular moment of this microscopic coupling: we retain the dominant nearest-Se contribution for each carbon atom, replace the magnitude of t_{zz} by the effective radial envelope $w(r) = e^{-\alpha r}$, and retain the bond direction $\hat{\boldsymbol{r}}_i = \boldsymbol{r}_i/|\boldsymbol{r}_i|$. Up to an overall hopping scale and the angular prefactor in Eq. (S10.4), this gives Eq. (S10.1). Accordingly, $\boldsymbol{d}_{pp}$ is not itself a hopping matrix element; it is a dimensionless, tunnelling-derived descriptor of the directional imbalance generated by a given registry. The effective parameter α controls how locally the descriptor samples the C–Se environment and is not fitted to the experimental anisotropy.

B. Orbital selectivity

The model isolates the interface-facing C p_z/Se p_z channel. For a predominantly out-of-plane C–Se bond, the factor $(z/r)^2$ in Eq. (S10.4) weights the σ-like contribution, whereas coupling from C p_z to Se p_x or Se p_y contains an additional in-plane direction cosine and is suppressed as the bond approaches the layer normal. This geometric argument motivates the p_z-only descriptor, but it does not imply that other Se orbitals or the Nb d states are absent from the full electronic structure. Such channels can change the absolute tunnelling strength and the detailed shape of the registry landscape. They cannot, however, create a preferred in-plane direction in an otherwise C_3-symmetric structure. The present model should therefore be interpreted as an orbital-resolved test of the short-range p_z registry mechanism; quantitative orbital weights and energy scales remain supplied by the DFT calculation.

C. Sensitivity to the decay constant α

We recomputed the sliding curves for $\alpha = 0.15$–$0.40\ \text{Å}^{-1}$ in both the commensurate and incommensurate constructions (Fig. S10.1). Changing α modifies the absolute magnitude and sharpness of $|\boldsymbol{d}_{pp}|$, as expected because it changes the spatial range of the weighting. The comparison between geometries is nevertheless stable: for every value tested, the $\sqrt{3} \times \sqrt{3}$ R30° ($\sqrt{3} \times \sqrt{3}$) curve displays stronger shift-dependent modulation and exceeds the 2×2 (2×2) curve over broad intervals. The conclusion of the model is therefore the relative registry sensitivity of the two geometries, rather than an absolute tunnelling amplitude at a particular α.

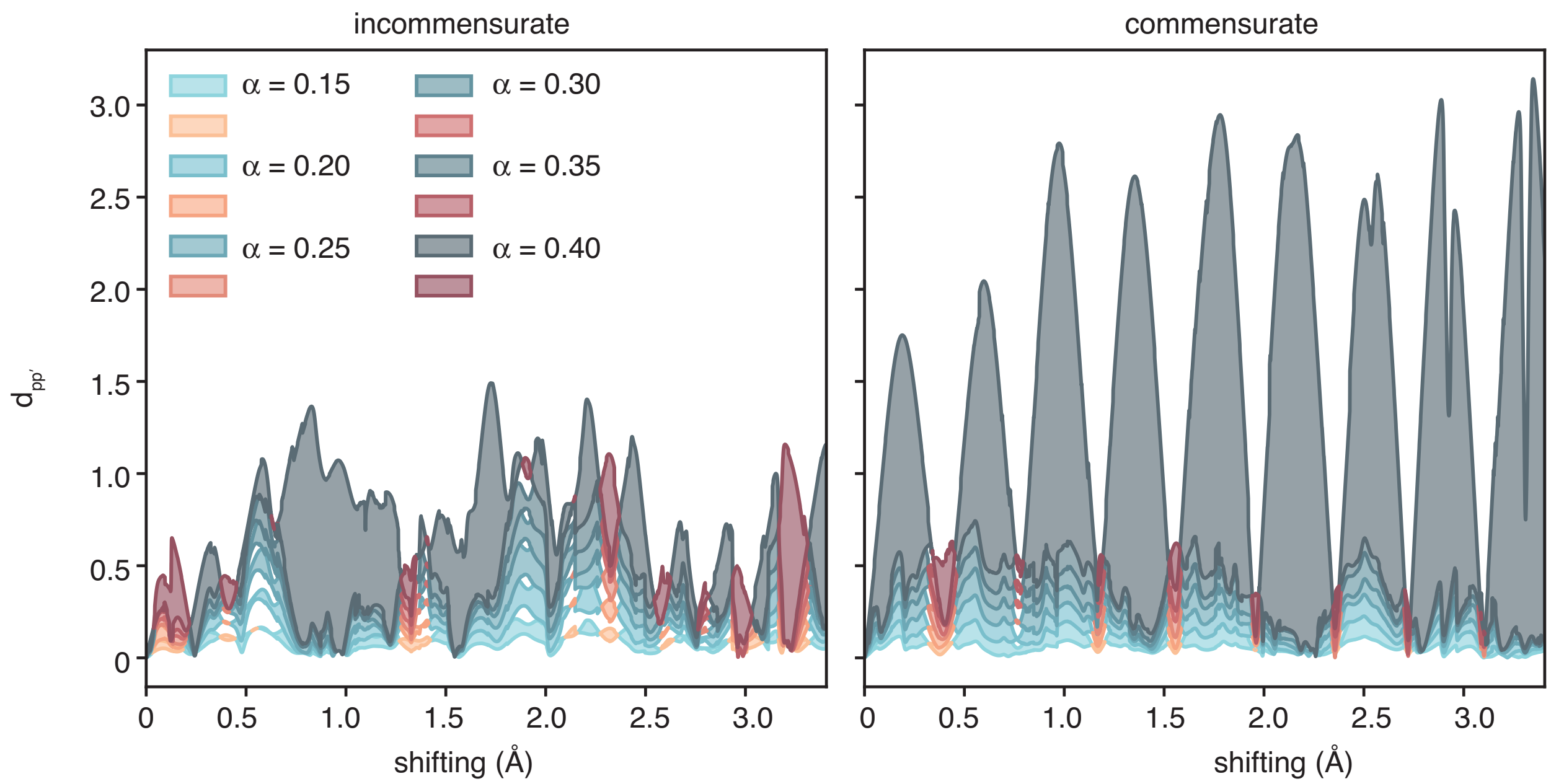

FIG. S10.1. **Magnitude $|\boldsymbol{d}_{pp}|$ as a function of lateral shift for the incommensurate (left) and commensurate (right) constructions.** The ordinate label $d_{pp'}$ in the plotted data denotes $|\boldsymbol{d}_{pp}|$, with p and p' referring to the C and Se orbitals, respectively. Curves are shown for the 2×2 and $\sqrt{3} \times \sqrt{3}$ geometries at $\alpha = 0.15$–$0.40\,\text{Å}^{-1}$. The shading indicates which geometry has the larger value at each shift. Although α changes the absolute magnitude and sharpness of the curves, the stronger registry dependence of the $\sqrt{3} \times \sqrt{3}$ geometry persists throughout the tested range.

D. Symmetry of the sliding map and its relation to C_3 breaking

By construction, the unshifted heterostructure is C_3 symmetric for both the 2×2 and $\sqrt{3} \times \sqrt{3}$ R30° geometries, and the full sliding map $\boldsymbol{d}_{pp}(\boldsymbol{v})$ inherits this symmetry. A C_3 rotation centre is visible, for example, near $\boldsymbol{v} \approx (-0.4,\, 0.2)$ in Fig. S10.2a; it need not coincide with the plotted origin because translation is periodic in the graphene lattice. The three 120°-related directions are therefore equivalent. The model neither imposes a preferred direction nor selects a symmetry-broken state; it compares the response of the interlayer coupling to symmetry-lowering displacements.

For the 2×2 geometry, $|\boldsymbol{d}_{pp}|$ depends only weakly on the sliding vector. For the $\sqrt{3} \times \sqrt{3}$ R30° geometry, it varies strongly, indicating a larger electronic response to lateral sliding, heterostrain, or structural relaxation. This comparison identifies a microscopic route for amplifying a weak symmetry-lowering perturbation, but it does not establish the associated energy gain or equilibrium

displacement. The folding pathway (Γ- versus K-folding) enters only indirectly: folding and registry sensitivity are both fixed by the commensuration geometry, but folding is not itself the cause of the symmetry lowering.

We next repeated the sliding analysis using the incommensurate graphene lattice constants. Without exact periodicity, $\boldsymbol{d}_{pp}$ no longer vanishes at any sliding position because the bond directions do not cancel perfectly. The qualitative contrast nevertheless persists: the 2×2 geometry (Fig. S10.2d) shows weak sliding dependence, whereas the $\sqrt{3}\times\sqrt{3}\,\mathrm{R}30^\circ$ geometry (Fig. S10.2e) retains pronounced modulation. The line cuts in Fig. S10.2f confirm that the peak-to-valley variation remains substantially larger for the $\sqrt{3}\times\sqrt{3}\,\mathrm{R}30^\circ$ case. The enhanced registry sensitivity is therefore retained when exact commensurability is removed. This incommensurate calculation extends the comparison beyond the periodic supercells accessible to the present DFT calculations.

To verify that this contrast between the two geometries is not an artefact of the commensurate approximation, we repeated the sliding analysis using the incommensurate graphene lattice constants. In the incommensurate case, the absence of exact periodicity means that $\boldsymbol{d}_{pp}$ no longer vanishes at any sliding position, as the cancellation of bond directions is never perfect. Nevertheless, the qualitative difference between the two configurations persists: the 2×2 geometry (Fig. S10.2d) shows a relatively flat hybridisation with weak sliding dependence, while the $\sqrt{3}\times\sqrt{3}\,\mathrm{R}30^\circ$ geometry (Fig. S10.2e) retains pronounced modulations. The line cuts in Fig. S10.2f confirm that the ratio of peak-to-valley variation remains substantially larger for the $\sqrt{3}\times\sqrt{3}\,\mathrm{R}30^\circ$ case. This robustness against lattice relaxation from exact commensurability supports the conclusion that the enhanced sensitivity to lateral shifts and hence the propensity for C_3symmetry breaking is an intrinsic geometric property of the $\sqrt{3}\times\sqrt{3}\,\mathrm{R}30^\circ$ superlattice, rather than a consequence of enforcing commensurability in the model.

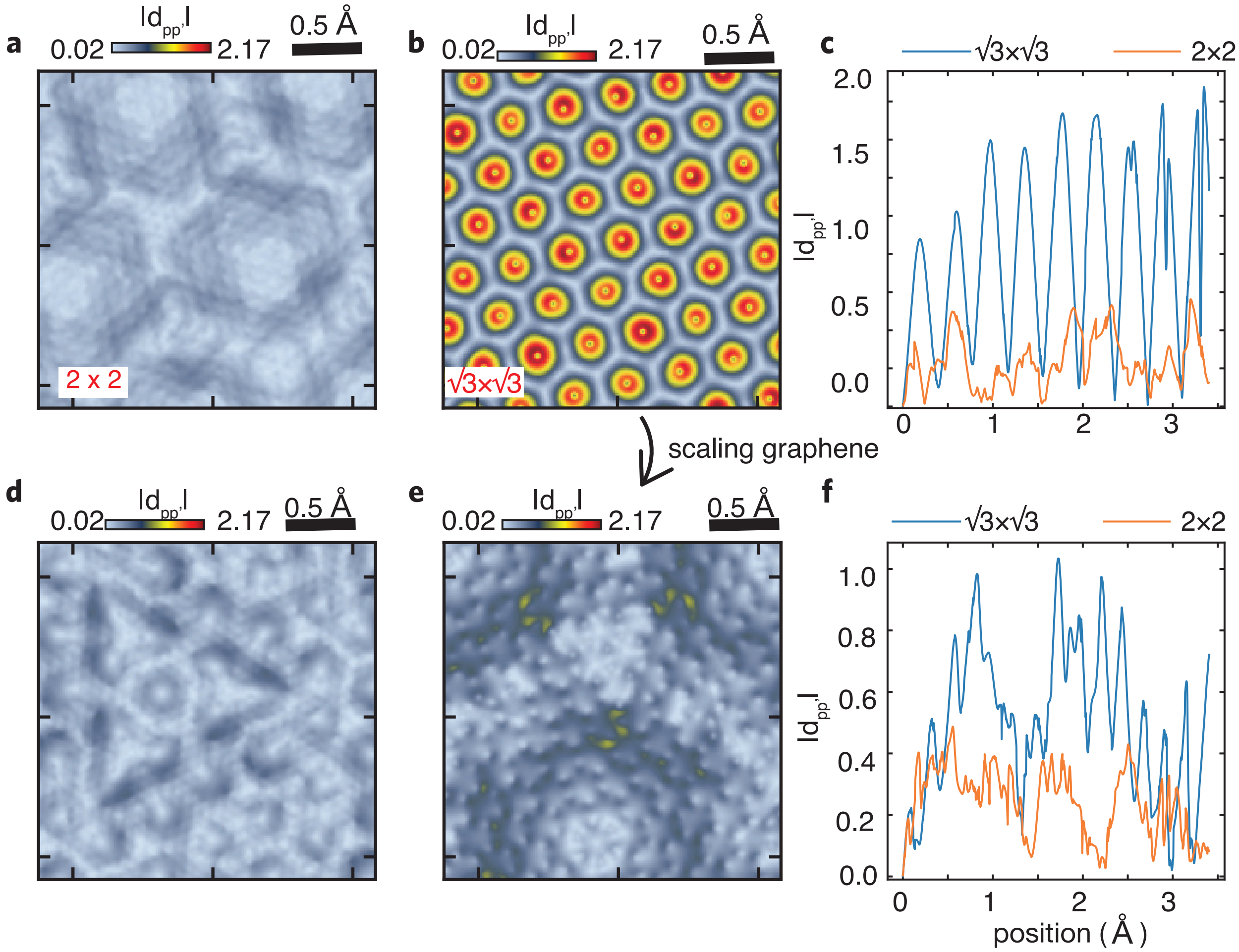

FIG. S10.2. **Qualitative illustration of geometric symmetry breaking based on a tight-binding-like estimate of average bond direction.** **a,** 2D resolved sliding map of the calculated hybridisation magnitude $|d_{pp}|$ for the commensurate 2×2 superlattice. (same dataset as Fig. 4b of the main paper). For each panel, the x and y axes refer to sliding the top graphene layer with respect to the bottom $NbSe_2$ by a vector $\vec{v} = (x, y)$. The colour quantifies the average oriented distance between nearest-neighbour C/Se p_z orbitals. **b,** 2D resolved sliding map of the calculated hybridisation magnitude $|d_{pp}|$ for the commensurate $\sqrt{3}\times\sqrt{3}$ R30° superlattice (same dataset as Fig. 4c of the main paper). **c**, Comparison of the $|d_{pp}|$ as a function of sliding between these two commensurate configurations (same dataset as Fig. 4d of the main paper). **d,** 2D resolved sliding map of the calculated hybridisation magnitude $|d_{pp}|$ for the 2×2 superlattice in the incommensurate superlattice configuration. **e,** 2D resolved sliding map of the calculated hybridisation magnitude $|d_{pp}|$ for the $\sqrt{3}\times\sqrt{3}$ R30° superlattice in the incommensurate superlattice configuration. **f,** Comparison of the $|d_{pp}|$ as a function of sliding between these two incommensurate configurations.

S11. OPTICAL IMAGES OF THE DEVICE

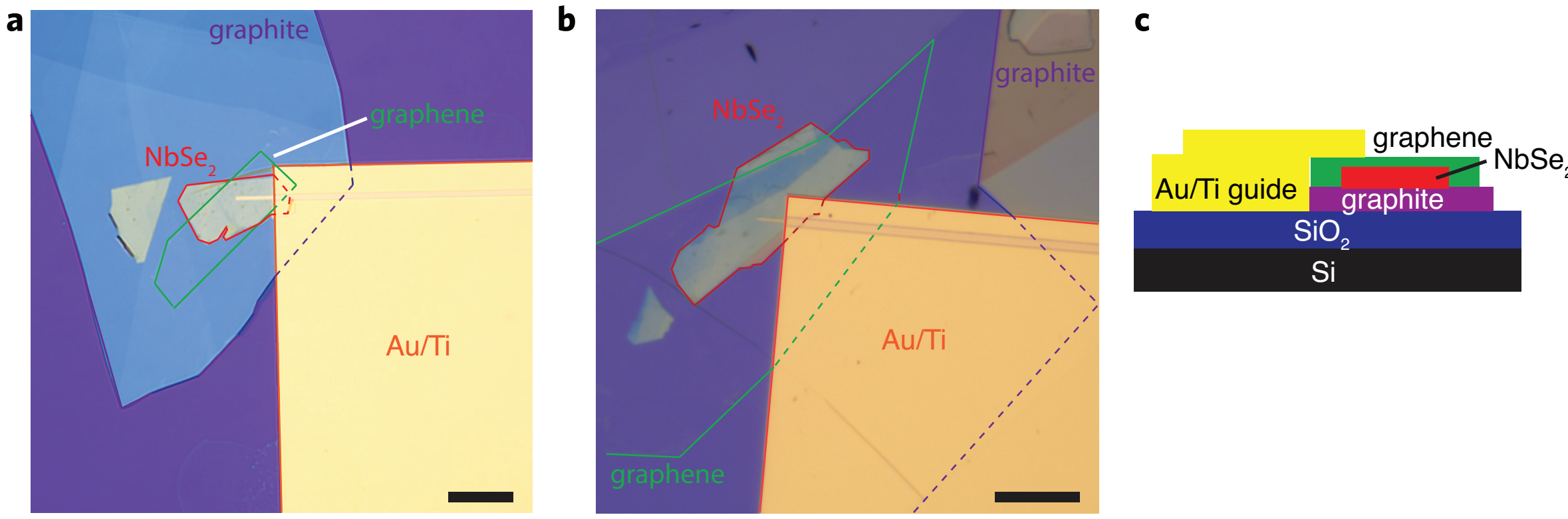

FIG. S11.1. **Sample overview.** **a,** Optical image of the $9°$ device composed of graphene (green line) on top of $NbSe_2$ (red line) on graphite (dark blue line) fabricated by mechanical assembly. Two lithographic processes were performed to contact and guide the STM tip towards the heterostructure gold (region). Scale bar: $20\,\mu m$. **b,** Same as a, but for the $27°$ device. **c,** Schematic side view of the devices.

S12. TOPOGRAPHY OF THE TWO SAMPLES BEFORE THE NANOSCALE PHASE ENGINEERING

We performed low-bias STM topography on the graphene/2H-$NbSe_2$ heterostructures prior to bias pulsing. In agreement with Chen et al. [15], the topographies of Gr/2H-$NbSe_2$ clearly show graphene, $NbSe_2$ lattices, and the moiré superlattices. However, the characteristic 3×3 charge density wave modulation of pristine 2H–$NbSe_2$ was not observed.

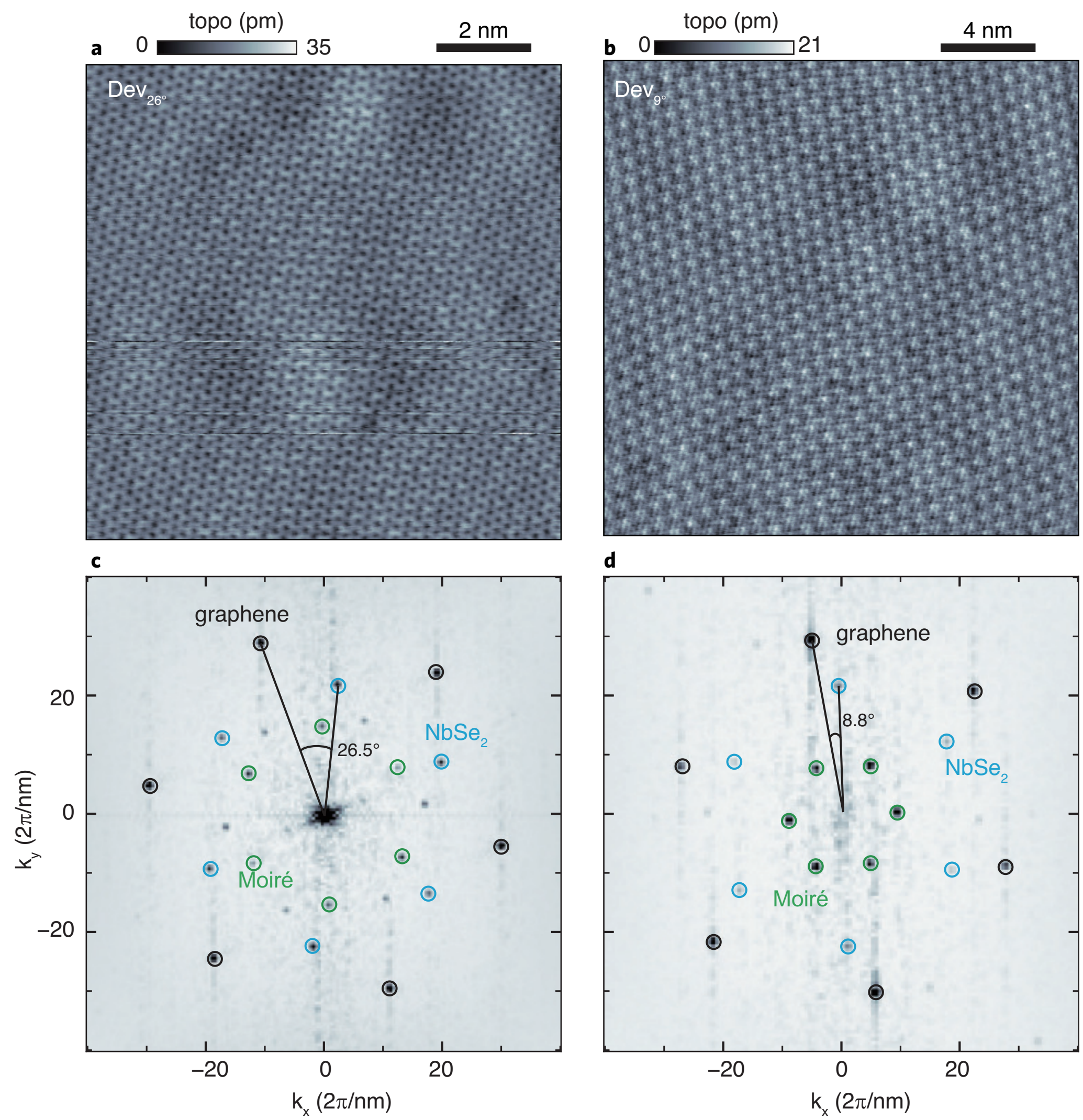

FIG. S12.1. **Atomically resolved STM topographical images a,** STM topographical image of the 26° device ($V = 300$ mV, $I = 200$ pA). **b,** STM topographical image of the 9° device ($V = 50$ mV, $I = 100$ pA). **c,d,** The corresponding FFT images.

S13. NANOSCALE ENGINEERING TO GENERATE GRAPHENE/1T-NBSE$_2$ SAMPLES

To build the target structure of graphene stacked on 1T-NbSe$_2$, we applied voltage pulses ranging from 2 to 5 V across the STM tip-sample junction. This procedure induces the structural transition of 2H-NbSe$_2$ underneath the graphene, resulting in the graphene being placed atop a single layer of NbSe$_2$ in the 1T phase on bulk 2H-NbSe$_2$.

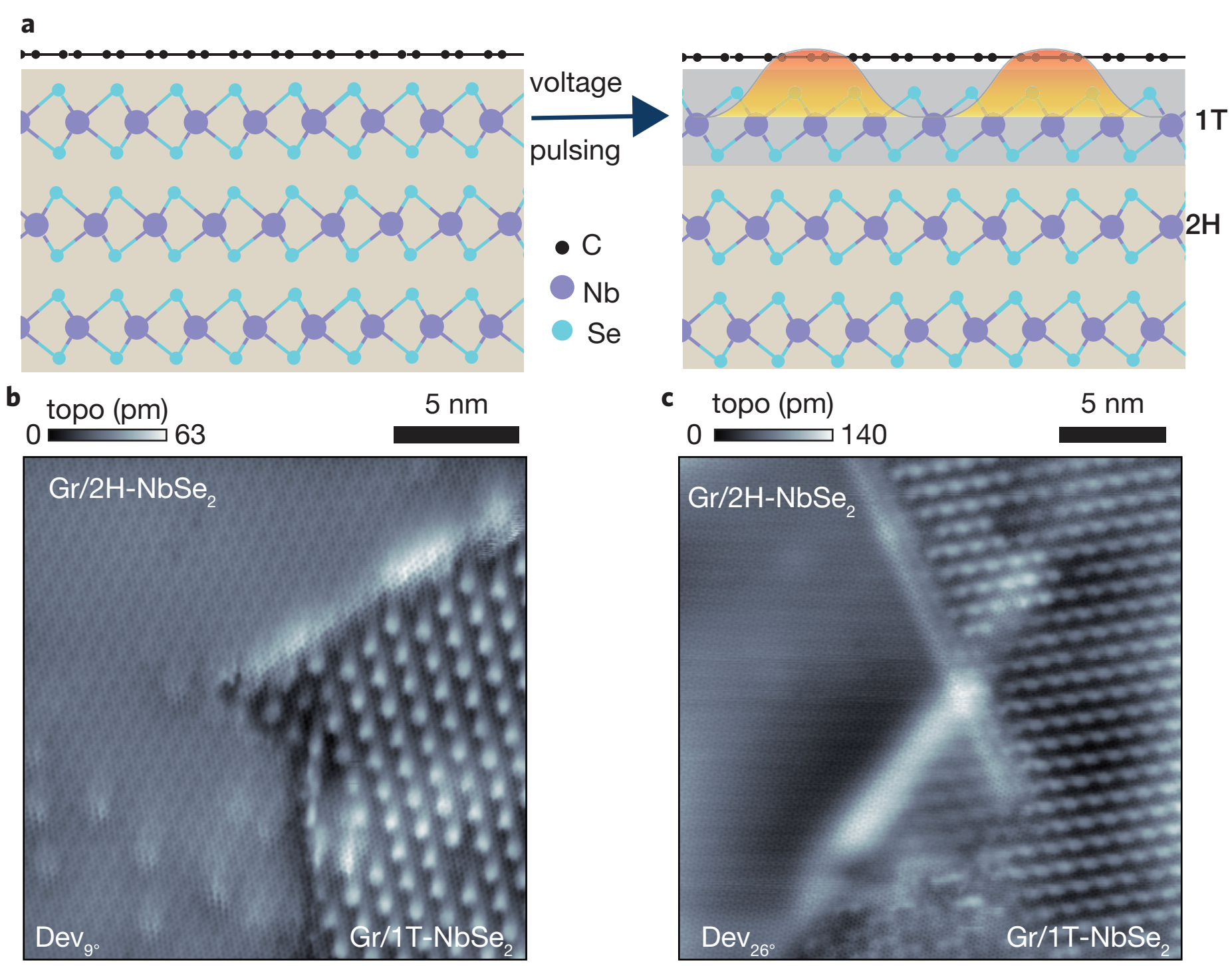

FIG. S13.1. **Phase transition from Gr/2H-NbSe$_2$ to Gr/1T-NbSe$_2$. a,** Schematical side view showing the vertical structure before and after voltage pulsing. **b,** STM topography of the phase boundary between graphene/2H-NbSe$_2$ and graphene/1T-NbSe$_2$/2H-NbSe$_2$ of the sample with a twist angle of 9° (2×2) (V = 650 mV, I = 100 pA). **c,** Same as b for the sample with a twist angle of 27° $(\sqrt{3} \times \sqrt{3}\ \mathrm{R30°})$ (V = 300 mV, I = 200 pA).

.

S14. DETAILS OF THE DFT CALCULATIONS

To generate the commensurate atomistic heterostructures between 1T-$NbSe_2$ and graphene to perform DFT calculations on, we started by fixing the 1T-$NbSe_2$ lattice parameters to $\boldsymbol{a}_1 = (8.9504,\ 8.6125)$ Å and $\boldsymbol{a}_2 = (-11.9338,\ 3.4450)$ Å to generate the $\sqrt{13} \times \sqrt{13}$ R13.9°, i. e., the Star-of-David CDW phase. A commensurate graphene 2×2 and $\sqrt{3} \times \sqrt{3}$ R30° superlattice of graphene on 1T-$NbSe_2$ was then constructed by straining graphene homoaxially and twisting its lattice vectors, while the 1T-$NbSe_2$ lattice and its $\sqrt{13} \times \sqrt{13}$ R13.9° CDW were kept at their relaxed lattice constants . The graphene lattice vectors are $\boldsymbol{a}_3 = (\sqrt{3}a_0/2, a_0/2)$ and $\boldsymbol{a}_4 = (\sqrt{3}a_0/2, -a_0/2)$. For the 2×2 structure, we found $a_0 \approx 2.35$ Å, $n = 11$, $m = -1$ and $\theta = 9.15°$, while for the $\sqrt{3} \times \sqrt{3}$ R30° structure, we found $a_0 \approx 2.42$ Å, $n = 3, m = 7$ and $\theta = 26.92°$. These lattice vectors provided an approximate structure to start the DFT calculations. Here, we note that for graphene, the required compression can rescale hopping amplitudes and Fermi velocities, but it preserves the full C_3symmetry of the supercell and the Dirac character of the bands. It therefore cannot by itself seed a directional C_3-breaking field. The remaining question is whether the enforced periodicity of the commensurate approximation changes the registry landscape; this is tested with the incommensurate evaluation of the geometric model in Sec. S10 (Supplementary Fig. S10.2d–f). Structural models of the two supercells, with the local stacking sites labelled, are shown in Supplementary Fig. S14.1.

To perform the DFT calculations, we utilised the Quantum Espresso code [16]. All calculations were performed using the PBE exchange-correlation functional [17] and a D3 van der Waals correction, ultrasoft pseudopotentials [18], the cold smearing scheme with a Gaussian smearing of 0.005 Ry, a 2D cutoff (c lattice parameter set of 20 Å), and the kinetic energy cutoff for wavefunctions of 40 Ry and charge density of 400 Ry. The $\boldsymbol{k}$-point grids utilised were different for the two investigated supercells, so these will be specified each time, but otherwise the settings were kept consistent.

The 2×2 and $\sqrt{3} \times \sqrt{3}$ R30° models are the smallest periodic cells that are commensurate with graphene and reproduce the experimentally observed 2×2 and $\sqrt{3} \times \sqrt{3}$ R30° repeats of the $\sqrt{13} \times \sqrt{13}$ R13.9° CDW, respectively. Across one moiré period, each cell samples the spatially varying local alignment of graphene with the CDW; the construction therefore does not represent the entire interface by a single chosen high-symmetry stacking. No additional registry constraint was imposed, and the relative translation, interlayer separation, and atomic corrugation were al-

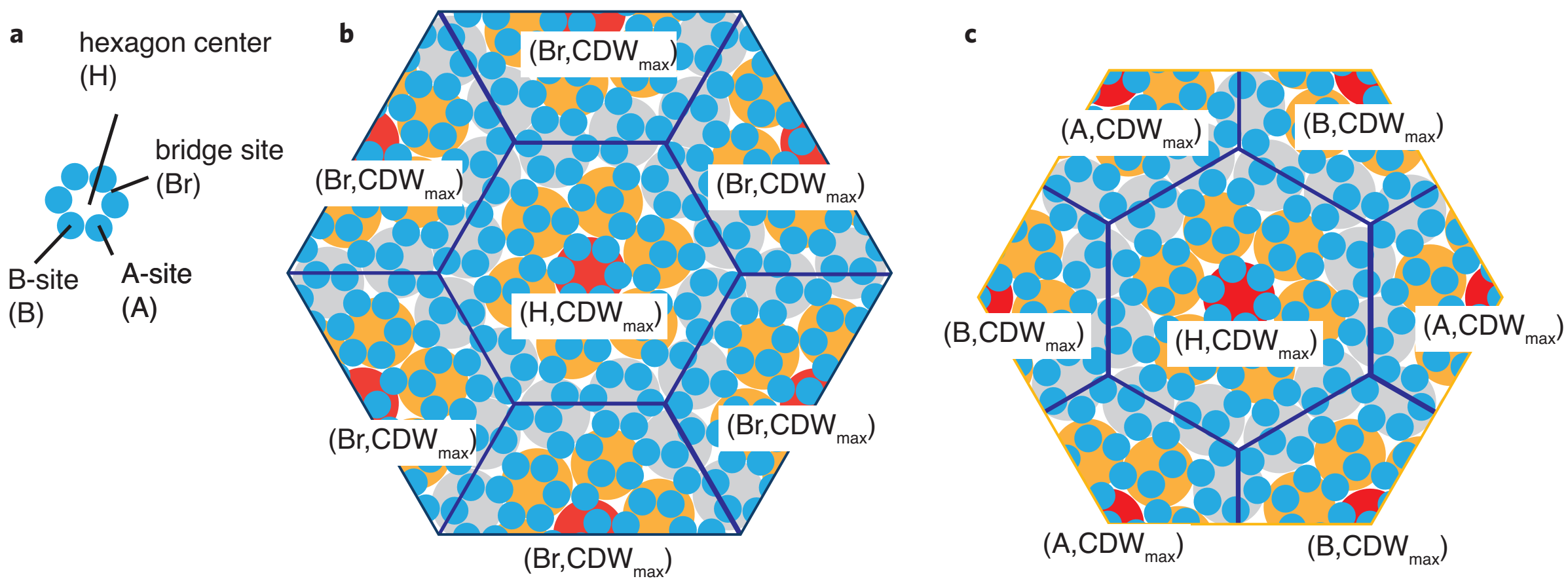

FIG. S14.1. **Structural models of the 2×2 and $\sqrt{3} \times \sqrt{3}$ R30° supercells and their local stacking registries complementary to the main-text Fig. 1d,f. a,** Definition of the high-symmetry stacking sites of graphene. **b,** The local stacking registries of 2×2 supercell. **c,** The local stacking registries of $\sqrt{3} \times \sqrt{3}$ R30° supercell.

lowed to adjust during relaxation. The relaxed models reproduce the registry multiplicities seen experimentally: the CDW maxima have nearly equivalent local environments in the 2×2 structure (main-text Fig. 1g), whereas three inequivalent local environments repeat in the $\sqrt{3} \times \sqrt{3}$ R30° structure (main-text Fig. 1i).

Initially, a pristine $\sqrt{13} \times \sqrt{13}$ R13.9° cell of 1T-$NbSe_2$ was relaxed using BFGS with a fixed cell and default settings, using a $4 \times 4 \times 1$ $\boldsymbol{k}$-point grid. The 1T-$NbSe_2$ spontaneously forms a $\sqrt{13} \times \sqrt{13}$ R13.9° CDW phase, characterised by, amongst a number of features, in-plane periodic lattice distortions of the Nb atoms. This CDW unit cell was then used to generate the 2×2 and $\sqrt{3} \times \sqrt{3}$ R30° superlattice cells, which are approximately commensurate with graphene. Similarly, the initial graphene structures of these structures were generated and relaxed, using default settings and a $\boldsymbol{k}$-point grid of $3 \times 3 \times 1$. Finally, to generate the graphene/1T-$NbSe_2$ superlattices, in the 2×2 and $\sqrt{3} \times \sqrt{3}$ R30° supercells, these atomic positions were combined where the graphene layer was displaced in z by $5\,\text{Å}$ relative to the plane of Nb atoms. These structures were then relaxed, again using default settings, with Γ-point sampling and $3 \times 3 \times 1$ $\boldsymbol{k}$-points for the 2×2 and $\sqrt{3} \times \sqrt{3}$ R30° structures, respectively.

Following these scf calculations, non-scf (nscf) calculations and post-processing to calculate the band structures, DOS and projected and LDOS were performed. For the 2×2 superlattice, a $6 \times 6 \times 1$ $\boldsymbol{k}$-point grid was calculated to obtain the DOS, and for the $\sqrt{3} \times \sqrt{3}$ R30° superlattice, a

$9 \times 9 \times 1$ $\boldsymbol{k}$-point grid for the nscf was calculated to obtain the DOS. From these finer nscf $\boldsymbol{k}$-point grids, the LDOS was calculated with an energy smearing of $10\,\mathrm{meV}$ calculated with a grid spacing of $10\,\mathrm{meV}$. The atom-resolved projected DOS was calculated by summing up all the contributions from the different atomic orbitals. To produce the LDOS pictures shown in the main text, we convolved the z contributions using an exponential function starting from a position $3\,\text{Å}$ above the graphene using a decay length of $3\,\text{Å}$. The LDOS was then additionally smeared in-plane using a Gaussian smearing scheme with a $\sigma = 2.46\,\text{Å}$. This calculation was performed on a unit cell, taking into account periodic images, and replicated. The band structure was also resolved in terms of the orbital character by printing the $\boldsymbol{k}$-resolved projected DOS, and using the p_z character in a Fermi function, with appropriately chosen "Fermi energy" and "temperature" to demarcate if a band was graphene (C p_z) or 1T-$NbSe_2$.

We do note, however, that while the DFT calculations do not have exact quantitative agreement with the experiments, the DFT calculations have largely captured the qualitative differences in the structures, and the differences are more down to exact energy orderings.

---

[1] S. Günther, P. Zeller, B. Böller, and J. Wintterlin, Chemphyschem **22**, 870 (2021).

[2] E. A. Wood, J. Appl. Phys. **35**, 1306 (1964).

[3] C. Rubio-Verdu, S. Turkel, Y. Song, L. Klebl, R. Samajdar, M. S. Scheurer, J. W. F. Venderbos, K. Watanabe, T. Taniguchi, H. Ochoa, L. Xian, D. M. Kennes, R. M. Fernandes, A. Rubio, and A. N. Pasupathy, Nat. Phys. **18**, 196 (2022), 2009.11645.

[4] A. Kerelsky, L. J. McGilly, D. M. Kennes, L. Xian, M. Yankowitz, S. Chen, K. Watanabe, T. Taniguchi, J. Hone, C. Dean, A. Rubio, and A. N. Pasupathy, Nature **572**, 95 (2019).

[5] G. Gruner, *Density waves in solids* (CRC press, 2018).

[6] S. Edkins, *Visualising the charge and Cooper-pair density waves in cuprates* (Springer, 2017).

[7] K. Rossnagel, J. Phys.: Condens. Matter **23**, 213001 (2011).

[8] A. Pasztor, A. Scarfato, M. Spera, C. Barreteau, E. Giannini, and C. Renner, Phys. Rev. Res. **1**, 033114 (2019).

[9] A. Pasztor, A. Scarfato, M. Spera, F. Flicker, C. Barreteau, E. Giannini, J. v. Wezel, and C. Renner, Nat. Commun **12**, 6037 (2021).

[10] K. P. Nuckolls, R. L. Lee, M. Oh, D. Wong, T. Soejima, J. P. Hong, D. Calugaru, J. Herzog-Arbeitman,

B. A. Bernevig, K. Watanabe, T. Taniguchi, N. Regnault, M. P. Zaletel, and A. Yazdani, Nature **620**, 525 (2023).

[11] E. Kamil, J. Berges, G. Schönhoff, M. Rösner, M. Schüler, G. Sangiovanni, and T. O. Wehling, J. Phys.: Condens. Matter **30**, 325601 (2018).

[12] J. C. Slater and G. F. Koster, Phys. Rev. **94**, 1498 (1954).

[13] G. Trambly De Laissardière, D. Mayou, and L. Magaud, Nano Lett. **10**, 804 (2010).

[14] P. Moon and M. Koshino, Phys. Rev. B **85**, 195458 (2012).

[15] Y. Chen, L. Wu, H. Xu, C. Cong, S. Li, S. Feng, H. Zhang, C. Zou, J. Shang, S. A. Yang, K. P. Loh, W. Huang, and T. Yu, Adv. Mater. **32**, 2003746 (2020).

[16] P. Giannozzi, O. Andreussi, T. Brumme, O. Bunau, M. B. Nardelli, M. Calandra, R. Car, C. Cavazzoni, D. Ceresoli, M. Cococcioni, N. Colonna, I. Carnimeo, A. D. Corso, S. d. Gironcoli, P. Delugas, R. A. D. Jr, A. Ferretti, A. Floris, G. Fratesi, G. Fugallo, R. Gebauer, U. Gerstmann, F. Giustino, T. Gorni, J. Jia, M. Kawamura, H.-Y. Ko, A. Kokalj, E. Küçükbenli, M. Lazzeri, M. Marsili, N. Marzari, F. Mauri, N. L. Nguyen, H.-V. Nguyen, A. Otero-de-la Roza, L. Paulatto, S. Poncé, D. Rocca, R. Sabatini, B. Santra, M. Schlipf, A. P. Seitsonen, A. Smogunov, I. Timrov, T. Thonhauser, P. Umari, N. Vast, X. Wu, and S. Baroni, J. Phys.: Condens. Matter **29**, 465901 (2017).

[17] J. P. Perdew, K. Burke, and M. Ernzerhof, Phys. Rev. Lett. **77**, 3865 (1996).

[18] K. F. Garrity, J. W. Bennett, K. M. Rabe, and D. Vanderbilt, Comput. Mater. Sci. **81**, 446 (2014).
